# Insights Into the Nature of Quantum Emitters in Electron‐Irradiated Hexagonal Boron Nitride

**DOI:** 10.1002/advs.77322

**Published:** 2026-08-31

**Authors:** Mouli Hazra, Anna Rupp, Mohammad N. Mishuk, Josefine Krause, Anand Kumar, Julien Chénedé, Mingi Kang, Bayarjargal N. Tugchin, Marijn Rikers, Isabelle Staude, Thomas Pertsch, Alexander Högele, Tobias Vogl

**Affiliations:** ^1^ Department of Computer Engineering, TUM School of Computation, Information and Technology Technical University of Munich Munich Germany; ^2^ Munich Center for Quantum Science and Technology (MCQST) Munich Germany; ^3^ Fakultät für Physik, Munich Quantum Center, and Center for NanoScience (CeNS) Ludwig‐Maximilians‐Universität München Munich Germany; ^4^ Max Planck School of Photonics Jena Germany; ^5^ Institute of Applied Physics, Abbe Center of Photonics Friedrich Schiller University Jena Jena Germany; ^6^ Institute of Solid State Physics Friedrich Schiller University Jena Jena Germany; ^7^ ARC Centre of Excellence for Transformative Meta‐Optical Systems, Department of Quantum Science and Technology, Research School of Physics Australian National University Canberra Australia; ^8^ Fraunhofer‐Institute for Applied Optics and Precision Engineering IOF Jena Germany

**Keywords:** 2D materials, annealing, hyperspectral mapping, organic processing residue, quantum technologies, vertical localization

## Abstract

Quantum emitters in hexagonal boron nitride (hBN) have emerged as a promising solid‐state platform for quantum technology applications. However, a persistent challenge in the field is the unclear origin of many observed emission lines, particularly in the visible range, which can be difficult to distinguish from signals arising from organic or process‐induced contamination during sample preparations and handling. This ambiguity limits both the reproducibility of emitter generation and the reliable identification of truly intrinsic quantum defects. This work provides a step‐by‐step framework to assess whether quantum emitters in electron‐irradiated hBN are associated with organic contaminants introduced during sample preparation. We employ hyperspectral imaging, thermal annealing, and oxygen plasma etching to investigate the origin of the green‐yellow emitters in electron‐irradiated hBN. The combined results not only rule out organic contamination as the source of emission but also provide insight into the spectral variability, thermal stability, and vertical localization of the emitters generated in electron‐irradiated hBN that was created without any pre‐ or post‐processing. In addition, our experiments demonstrate the feasibility of creating stable emitters in hBN with thicknesses below 10 nm. These findings provide practical guidance for the identification and controlled implementation of hBN‐based single‐photon emitters in quantum photonic devices.

## Graphical abstract

Hyperspectral imaging is used to identify quantum emitters hosted by the 2D material hexagonal boron nitride. By selecting specific emission wavelengths, one can distinguish individual emitters from ensembles and gain important insights into the atomic structure of these emitters.

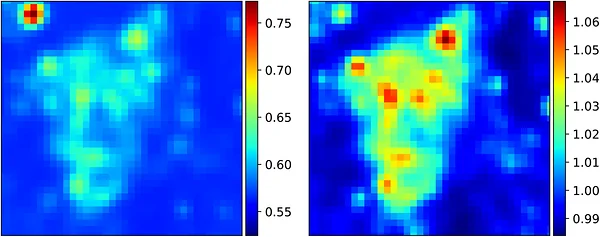

## 1 Introduction

Hexagonal boron nitride (hBN) provides a distinctive set of material advantages in scalable quantum technologies. Its wide bandgap [1] supports a diverse range of room temperature (RT) quantum emitters spanning from the near‐infrared to the ultraviolet [2, 3, 4, 5, 6]. Its two‐dimensional, atomically flat structure enables precise spatial control and seamless integration into on‐chip photonic architectures [7, 8, 9, 10]. This planar geometry not only simplifies fabrication processes but also improves optical accessibility, enhancing both excitation efficiency and photon collection [11, 12]. Beyond fundamental quantum physics experiments [13], demonstrated applications with hBN include sensing of temperature [14, 15, 16], pressure [17], strain [18], electric [19] and magnetic fields [17, 20, 21], as well as quantum cryptography [22], quantum computing [23], quantum random number generation [24] and space instrumentation [25, 26].

Despite these advancements, the practical deployment of hBN‐based quantum emitters remains limited by an incomplete understanding of their microscopic origin and the degree of control over their formation. These challenges are closely interconnected, as precise and reproducible emitter generation requires detailed knowledge of the underlying defect structures and their interaction with the local environment. To date, hBN emitters have exhibited substantial variability in their spectral characteristics [18, 27, 28, 29, 30] and lifetime [4, 18, 27, 31, 32, 33, 34, 35, 36, 37], with emission properties strongly influenced by strain [29, 38], temperature [14, 15, 16, 31, 39], electric [19, 40] and magnetic fields [17, 20, 41], and the surrounding dielectric environment [12, 42]. While this pronounced environmental sensitivity makes these emitters promising candidates for nanoscale sensing applications, it poses a challenge for quantum photonic technologies, where stable and reproducible emission is required. This variability has raised fundamental questions about the intrinsic versus extrinsic nature of the observed emission. In particular, several observations suggest that certain optical signatures attributed to hBN defects may instead originate from extrinsic sources, such as organic contaminants [43]. Several classes of organic molecules are known to exhibit photophysical properties remarkably similar to those reported for single photon emitters (SPEs) in hBN, including visible‐range emission, nanosecond‐scale lifetimes, pronounced vibronic sidebands, and high single‐photon purity [43, 44, 45, 46, 47, 48]. Given the wide bandgap of hBN [1], which can suppress non‐radiative quenching, the material can act as an efficient host matrix for such molecular emitters [49]. This has led to ongoing debate as to whether at least a subset of reported emitters arises from contamination rather than intrinsic lattice defects.

Yellow quantum emitters in electron‐irradiated hBN have been demonstrated to exhibit reproducible and stable single‐photon emission, with a characteristic emission peak around 575 nm at RT and high single‐photon purity [9, 34, 50]. At cryogenic temperatures, a well‐resolved zero phonon line (ZPL) emerges at 547.5 nm, indicating that these so‐called “yellow” emitters more precisely fall within the green–yellow spectral range [14]. However, as is the case for most hBN emitters reported so far, their microscopic origin remains elusive. In addition, low‐acceleration‐voltage electron‐beam irradiation is known to induce the deposition of a carbonaceous film originating from residual hydrocarbons on the sample surface and/or scanning electron microscope (SEM) chamber [51, 52]. There are several unavoidable sources of organic contamination during sample preparation. For instance, mechanical exfoliation using adhesive tape can leave glue residues; polymers are commonly used to transfer hBN flakes onto substrates. No pre‐ or post‐processing steps such as annealing, plasma or UV cleaning were performed in this case. While minimizing processing steps is advantageous in terms of simplicity and time efficiency, it also increases the likelihood of introducing emitters that are not intrinsic to the hBN lattice. This motivated a systematic investigation of the emitter origin. Rather than modifying the fabrication process, we performed a step‐by‐step analysis of the emission properties using a combination of experimental techniques.

First, we performed hyperspectral mapping on electron‐irradiated hBN to investigate any heterogeneity in the emitter creation process across the flake. These measurements were carried out at 4.5 K, 155 K, and RT, enabling us to examine (i) whether multiple emitter species are generated, (ii) how emitter formation correlates with the spatial location of electron beam exposure, and (iii) how emitters at individual spatial locations exhibit temperature‐dependent spectral behavior. Second, the electron‐irradiated hBN with green‐yellow emitters was subjected to incremental heat treatments in an inert environment. This approach is motivated by the fact that certain organic compounds can become fluorescent upon heating, while intrinsic defects in hBN gain mobility at temperatures above 800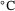 [33, 53, 54]. Organic molecules are generally sensitive to thermal treatment even at relatively low temperatures [45, 46, 47, 48]. Therefore, if organic molecules contribute to the observed green‐yellow emission, changes in emitter density or emission characteristics would be expected upon annealing. Finally, the vertical localization of emitter formation was investigated to clarify the potential role of contamination. Previous studies [43] have suggested that contamination‐related emitters in hBN are often located at the hBN/substrate interface. To probe this, we performed plasma etching on an electron‐irradiated hBN flake containing green–yellow emitters. The plasma progressively removed the hBN layers from the top surface, enabling us to determine the vertical localization of the emitters. A sub‐10 nm thick flake was intentionally selected to evaluate whether electron‐beam‐induced emitter formation is viable in ultrathin hBN. Based on these experimental results, we then evaluate the hypothesis of a contamination‐based origin and, more broadly, contribute to the understanding needed for the reliable implementation of hBN quantum emitters in future quantum technologies.

## 2 Results and Discussions

### 2.1 Hyperspectral Mapping of Electron‐Irradiated hBN

**FIGURE 1:**
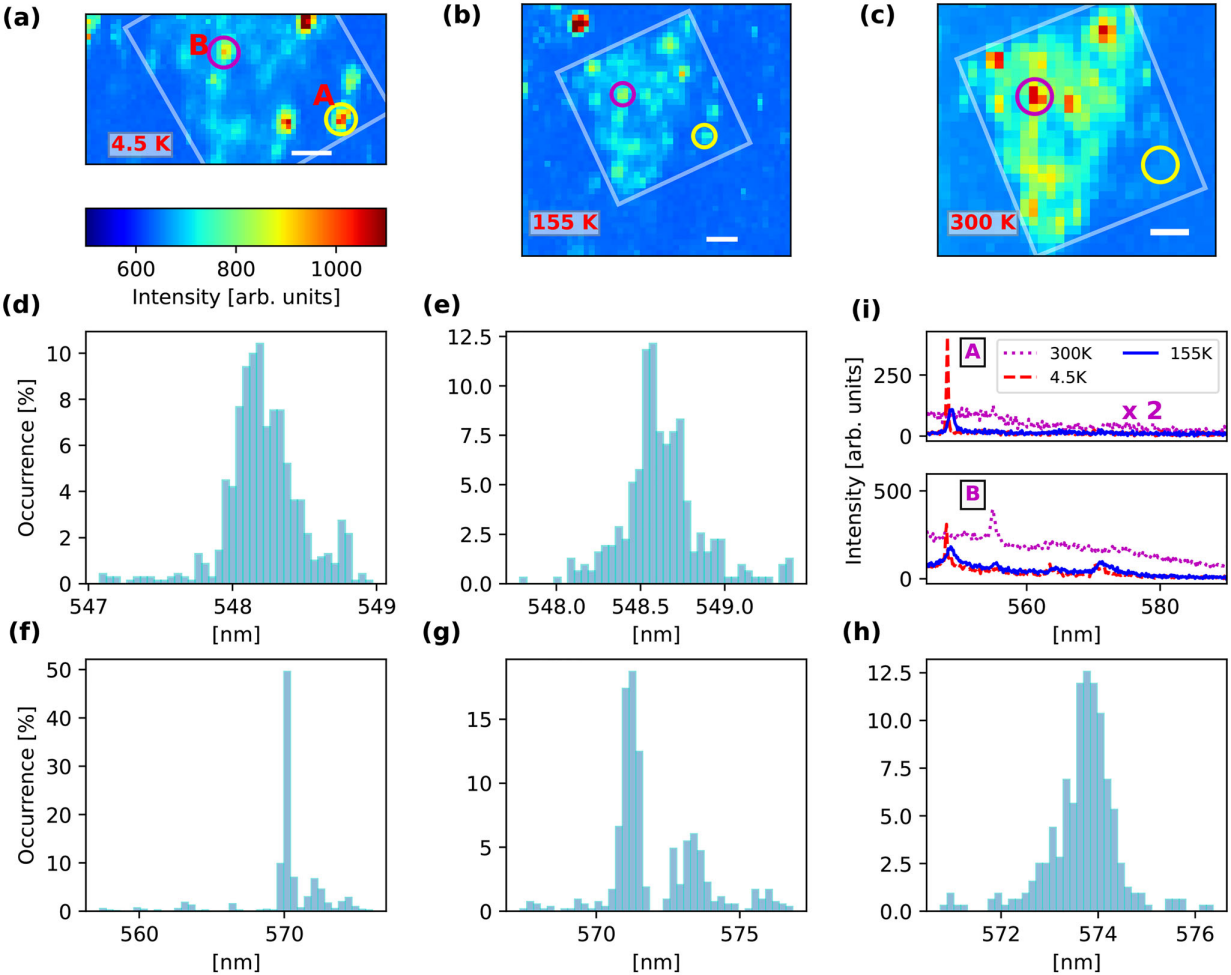
Hyperspectral images of electron‐irradiated hBN. (a–c) Intensity maps of ZPL‐filtered emission acquired with an excitation power of 180 μW at temperatures of 4.5 K, 155 K, and RT, respectively. The irradiated regions are marked by gray squares. The ZPL and PSB at each pixel were fitted using Lorentzian and Gaussian functions, respectively. The extracted peak positions were compiled into histograms for the ZPL (d–e) at 4.5 K and 155 K, and for the PSB (f–h) at 4.5 K, 155 K, and RT. The ZPL peaks are centered at 548.2 nm (4.5 K) and 548.5 nm (155 K), while the PSB peaks are observed at 570.3 nm (4.5 K), 571.3 nm (155 K), and 573.8 nm (RT), indicating a temperature‐dependent spectral shift. (i) Representative spectra from two spatial locations indicated in (a). Scale bars in (a–c) correspond to 1 μm.

Our approach to assess the spectral variability of emitter creation is raster scanning the flake and recording the spectra. This contains more information than a standard single‐photon detector scan, as it provides full spectral information at each spatial position across the region of interest. Later, it can be processed to emphasize individual parts of the spectra over broad spectral backgrounds. This is particularly important for irradiated hBN, where electron‐beam exposure simultaneously creates quantum emitters and carbonaceous deposits (see Figures S1–S3). When the full spectral range is integrated into a single intensity map, the resulting image is often dominated by the broadband luminescence from the carbonaceous layers, masking the spatial signature of individual emitters. In contrast, by integrating only a narrow spectral window around the characteristic peak of an emitter, the resulting intensity map reveals diffraction‐limited localized emitters, even when they are embedded in regions with strong background emission (see Figures S4 and S5). A squared area of 5 × 5 μm 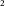 was irradiated in order to understand the spatial variability of emission spectra. Atomic force microscopy (AFM) measurements revealed a height increase, and low‐energy SEM imaging showed a darker contrast in the irradiated regions (see Figures S1–S3). These observations are consistent with electron‐beam‐induced deposition of a carbonaceous film originating from residual hydrocarbons in SEM environments [51, 52]. Hyperspectral maps at different temperatures zoomed around the ZPL in Figure 1a–c helped us visualize how the emission from spatially localized emitters evolves with temperature. We have performed a statistical analysis of ZPL and phonon sideband (PSB) emission peaks as shown in Figure 1d–h, respectively. This reveals the presence of a single class of emitter with ZPL centered around 548.2 nm and PSB near 570.3 nm at 4.5 K. With increasing temperature, they got redshifted and broadened (see Figures S6 and S7), consistent with previous reports [14] and generally attributed to the narrowing of the bandgap at elevated temperatures [39, 55, 56]. The ZPL emission variability is seen to be < 1 nm and the PSB peak shapes vary depending on the spatial location. As shown in Figure 1i, at some locations the PSB peaks are seen to be split, and their relative height is also seen to change. This advanced splitting has been observed in other hBN emitters before [57]. We speculate this as the domination of different phonon modes depending on the local environment. The electron–phonon coupling also varies among emitters, which influences their visibility at elevated temperatures. Emitters with weaker electron–phonon coupling (i.e., a weaker PSB, such as emitter A in Figure 1) become difficult to observe, as the RT emission is mostly dominated by the PSB. The ZPL at RT is strongly broadened and partially overlaps with the residual laser background and the hBN Raman (see Figure S4), preventing reliable extraction of peak features. For this reason, the RT ZPL was excluded from the statistical analysis. Notably, the emitters maintain high single‐photon purity at RT, even when the emission is only collected through PSB (see Figure S3). Therefore, we considered only PSB of the green‐yellow emitter for further investigations at RT, as evident in the later sections. Across the investigated temperature range, no additional distinct emitter species were observed within the spectral window of 532–600 nm. Overall, hyperspectral mapping validates the creation of a single class of emitters in electron‐irradiated hBN, with emitters predominantly localized within the irradiated region. We did not observe large variability of emission energy and spectral shape as typically observed in organic molecules and other reported hBN emitters.

### 2.2 Thermal Stability and Contamination Effects in Electron‐Irradiated hBN

**FIGURE 2:**
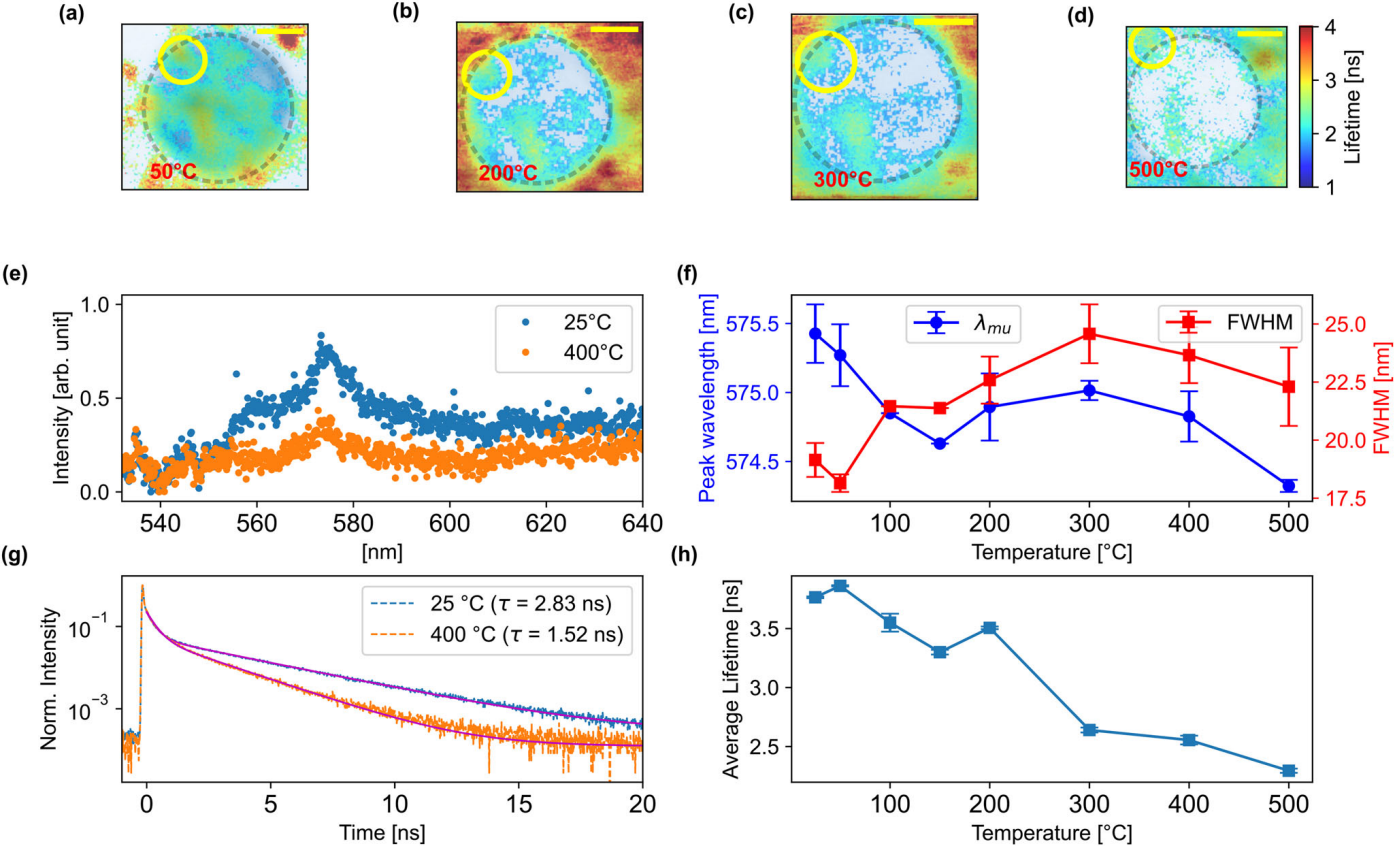
Photophysical characterization of emitters following 30 min annealing in an Ar atmosphere at different temperatures. (a–d) PL lifetime maps of a irradiated region (marked as grey dotted circle) after annealing at 50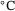, 200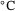, 300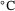, and 500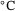, respectively. These maps reveal the emergence of thermally activated emitters outside the irradiated region and highlight a fundamental difference in emitter density between irradiated and non‐irradiated areas. (e) Representative PL spectra recorded at two different annealing temperatures. (f) Central wavelength and FWHM of the PSB as a function of annealing temperature. (g) Lifetime measured at two temperatures. (h) Average lifetime as a function of temperature. The measurements shown in (e) and (g) correspond to the emitter highlighted by the yellow circle in panels (a–d). Scale bars in (a–d) correspond to 1 μm.

In addition to the broad spectral variability, certain organic compounds are known to exhibit fluorescence upon thermal treatment [43, 44, 46]. Annealing under an inert environment, therefore, serves as a tool to investigate the presence of organic contamination in the emitter creation process. We annealed the electron‐irradiated hBN containing the green‐yellow emitters in an Ar environment in incremental temperature steps. The photophysical characteristics of the emitters were recorded for each annealing temperature after the sample cooled down to RT. This probes the thermal stability of green‐yellow emitters and checks if there is contamination present in the sample that gets thermally activated. The photoluminescence (PL) lifetime maps of a 3 μm‐wide circular irradiated region after annealing at different temperatures are shown in Figure 2a–d. At 50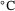, several green‐yellow emitters with PL lifetimes exceeding 3 ns are observed within the irradiated region. One representative emitter is highlighted by the yellow circle in the maps. To improve the visibility of the long‐lifetime emitters, pixels with lifetimes above 1 ns are displayed in the lifetime maps. The corresponding PL intensity maps are provided in Figure S8. At 50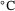 there is hardly any fluorescence outside the irradiated region. Upon annealing above 150 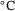, a high density of thermally activated emitters emerges uniformly throughout the non‐irradiated regions. But their density remains strongly suppressed within the irradiated area. These thermally activated emitters have a relatively higher lifetime compared to the green‐yellow emitter, as evident in Figure 2b,c. This behavior highlights a fundamental difference between emissions from irradiated and non‐irradiated regions. Further distinction arises from their photophysical properties: emission from the non‐irradiated areas is broad, undergoes photobleaching under prolonged laser excitation and fades away in high‐temperature annealing (see Figure S9). This indicates a contamination‐related origin [48]. In contrast, the green‐yellow emitters localized within irradiated regions exhibit stable emission without blinking or bleaching, as shown in Figure S10. Their emission spectra (Figure 2e,f) remain unchanged in the range of observed annealing temperatures up to 500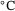. Usually at this temperature range, atomic reconstruction is improbable [33]. An unchanged spectrum also indicates that the underlying defect structure of the emitter remains unchanged. Moreover, the persistent suppression of contamination‐related fluorescence in irradiated regions suggests that electron irradiation alters the local environment in a way that prevents the activation of unwanted emitters. Notably, a gradual decrease in total lifetime and emission intensity was observed as shown in Figure 2g,h and Figure S10, respectively. This may arise from the activation of additional nonradiative decay channels upon annealing. Additionally, both spectral and lifetime measurements across the flake remain comparatively homogeneous at each annealing step, without evidence of strongly varying sub‐populations that might be expected from a distribution of independent contaminants with different thermal stabilities. The slight red‐shift in peak energy (∼ 1 nm) may be associated with a change in local environment or strain modification in the sample, although this requires further investigation. The observed narrowing of the full width at half maximum (FWHM) after high‐temperature annealing can be a fitting artifact. As the Gaussian fit may capture only the clearly resolved part of the spectrum, leading to an apparent narrowing. Alternatively, the narrowing may result from reduced inhomogeneous broadening due to partial cleaning during annealing.

The emitters are no longer detectable after annealing above 500 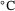. The irradiated regions appear significantly darker (see Figure S10), consistent with the removal of carbonaceous deposits and the associated suppression of broad background emission [50, 52]. The loss of detectable signal from the emitters is indicative of a substantial reduction in quantum efficiency, reflected in the gradual decrease in both lifetime and intensity with increasing annealing temperature. This is likely caused by changes in the local defect environment due to high‐temperature annealing. Notably, diffraction‐limited green–yellow emitter spots in the irradiated regions remain preserved up to 500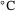, as highlighted by the yellow circles in PL maps in Figure 2a–d and Figure S8. This suggests that the emitters do not disappear progressively with annealing. If contamination were dominant, a broader distribution of thermal responses might be expected. Instead, emitters across the flake exhibit relatively homogeneous behavior in both spectral signature and lifetime, as well as in the eventual loss of optical signal.

Overall, these observations suggest that the defect structure remains intact with annealing up to 500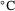, while the recombination dynamics are significantly affected. Together, our observations indicate that our samples are not free from contamination. But there is a clear distinction between thermally activated contamination fluorescence and the robust green‐yellow emitters, supporting a non‐contamination‐related origin for the latter. Besides, it also highlights an additional benefit of electron‐beam irradiation in suppressing unwanted background emission.

### 2.3 Vertical Localization of the Green‐Yellow Emitters

Beyond photophysical signatures, spatial localization provides an additional route to distinguish intrinsic emitters from contamination‐related origins. Hyperspectral mapping reveals that the green‐yellow emitters are deterministically generated at the electron‐beam irradiation sites. However, while this lateral correlation confirms their association with the irradiated regions, it does not reveal their vertical position within the hBN flake. Previous studies have suggested that contamination‐related emitters preferentially form at the hBN/substrate interface [43]. This motivates a direct investigation of the vertical localization of the emitters to determine whether they are located within the hBN crystal or at the hBN/substrate interface.

**FIGURE 3:**
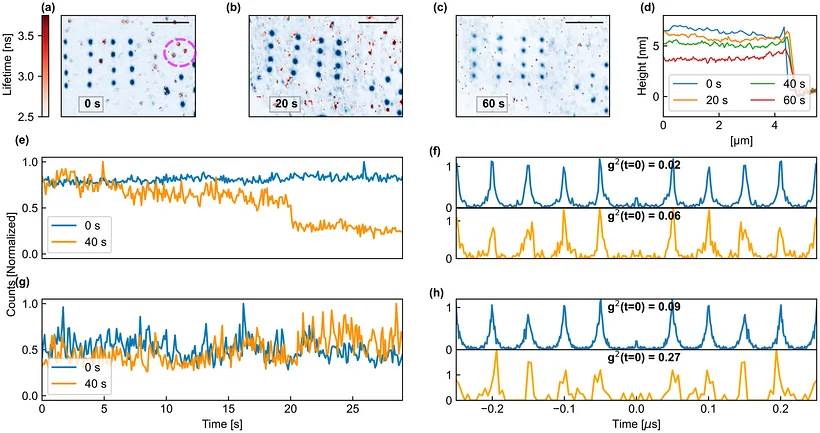
Layer‐by‐layer etching of an hBN flake. PL lifetime maps recorded (a) before, (b) after 20 s, and (c) after 60 s of oxygen plasma etching. Blue dots indicate the irradiated spots on the hBN flake, while red dots mark the emitters with 3–5 ns lifetime. The pink circle in (a) highlights emitters created far from the irradiated region. (d) hBN thickness function of plasma etching time. (e) PL intensity time trace of an emitter exhibiting photobleaching after 40 s of plasma etching, with the corresponding second‐order autocorrelation function, ($g^{\left(2\right)}\left(\tau \right)$), shown in (f) before (blue) and after (orange) etching. (g) PL intensity time trace of an emitter located in the thinnest region of the flake, confirming emitter creation in an hBN flake with an initial thickness of 6.4 nm. The emitter remains photostable after 40 s of plasma etching, corresponding to a reduced flake thickness of 5.2 nm. The corresponding ($g^{\left(2\right)}\left(\tau \right)$) measurements before (blue) and after (orange) etching are shown in (h). The scale bar in (a)–(c) is 5 μm.

To probe this, the irradiated hBN flake with green‐yellow emitters was thinned progressively using oxygen plasma while tracking the number of emitters after each etching step. PL lifetime maps depicted in Figure 3a–c give an overview of the emitter population as thinning of the flake occurs. The blue dots indicate the irradiated spots, and the red dots indicate emitters that have a lifetime of 3–5 ns. The thinning as a function of etching time is depicted in the AFM line profile in Figure 3d. Before any plasma etching, the green‐yellow emitters were identified and characterized. After the first 20 s etch, several new emitters appeared, visible as red spots in the lifetime map in Figure 3b. This is expected, as oxygen plasma itself can activate new luminescent defects in hBN [53]. Nevertheless, correlating the post‐etch confocal PL maps with the initial maps enabled us to identify the emitters that existed prior to plasma treatment and distinguish them from plasma‐induced emitters (as shown in Figures S11–S14). After an additional 20 s of etching, some of the green‐yellow emitters became unstable and photobleached during optical measurements, as shown in Figure 3e,f. This behavior indicates that the emitters were not yet physically removed; rather, removal of the upper hBN layers strongly affected their stability. Similar behavior has been reported for other hBN quantum emitters [42, 58], as emitter stability strongly depends on the surrounding crystal environment.

In Figure 3g, we demonstrate the time‐resolved PL counts from a green‐yellow emitter in a hBN flake of thickness 6.4 nm. When it was further etched to 5.2 nm, we still observed emission from the same emitter with single‐photon purity (Figure 3h), although the emission was weaker. The slightly higher ($g^{\left(2\right)}\left(0\right)$) value after etching is due to the reduced signal‐to‐noise ratio. Similar behavior for electron‐irradiated emitters (B‐centers) in very thin hBN has been reported previously [58]. The creation of emitters in such thin flakes has been correlated with electron scattering, where emitters can be found several microns away from the irradiated spots. We also observed emitters far from the irradiated spots, as depicted by the pink circle in Figure 3a and Figure S11, although this requires further investigation.

If the emitters were located at the hBN/substrate interface, they would be expected to persist irrespective of the hBN flake thickness. But we did not observe any green‐yellow emitters in flakes thinner than 5.2 nm. The irradiated regions still show bright emission in the PL map, but no green‐yellow emitters are found at this thickness, as shown in Figure S13. In the thicker parts of the flake, emitters are still present upon further thinning, as shown in Figures S11 and S12. Taken together, these results argue against an origin exclusively at the hBN/substrate interface. Instead, they show that the green‐yellow emitters are distributed within the hBN crystal while exhibiting a dependence on the surrounding environment.

### 2.4 Discussion

A simplified emitter fabrication process that omits cleaning steps introduces the risk of extrinsic contamination. Since many organic molecules can act as efficient visible single‐photon emitters, it is essential to carefully assess a possible extrinsic origin. In the experiments described above, we therefore compare key properties of organic emitters with those of green–yellow hBN emitters, namely spectral variability, response to thermal treatment, and spatial as well as vertical localization. The strong spatial correlation with electron beam irradiation suggests that any contamination‐related mechanism would also need to be beam‐induced. However, previous studies [59] have shown that similar irradiation conditions do not produce comparable emitters in other wide bandgap materials such as GaN, SiC, and mica, arguing against a generic contamination pathway. Furthermore, hyperspectral mapping reveals a well‐defined and narrow spectral distribution, in contrast to the broad and heterogeneous emission typically associated with contamination [44]. We also note the absence of spectral wandering or broad distributions of emission energy that are commonly observed for organic emitters. Spatially, the emitters are localized around the irradiated regions on the flake rather than at the flake edge, where organic molecules were reported previously in hBN [43]. In addition, plasma etching experiments reveal the persistence of emitters even after removal of several hBN layers from the top that is difficult to reconcile with a purely surface‐bound contaminant.

Annealing experiments provide further insight into the nature of these emitters. Both spectral and lifetime measurements across the flake remain homogeneous at each annealing step, with no evidence of a varying density of diffraction‐limited spots in the irradiated region up to 500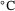. This is not necessarily expected for a distribution of independent contaminants with different thermal stabilities and binding environments. The loss of optical signal from the green–yellow emitters observed after annealing above 500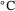 can, in principle, arise from several mechanisms; here, we speculate on two plausible scenarios. First, the emitters could be associated with interstitial‐type defects, which are expected to become mobile at comparatively lower temperatures than substitutional or vacancy defects, given that intrinsic lattice defects in hBN generally require higher temperatures (∼800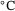) for migration [33, 60]. This can be further supported by the fact that the PSB appears at ∼90 meV from the ZPL [14], which is lower than the typical longitudinal optical (LO) phonon energy in hBN [61]. On the other hand, the out‐of‐plane optical (ZO) phonon mode has been reported at similar energies in interstitial defects [62]. One can also expect changes in emitter characteristics with increasing annealing temperature in such a case. Although a ∼1 nm shift in the PSB spectrum is observed at RT, this can also arise from spectral diffusion or changes in the local dielectric or strain environment. Alternatively, the emitters may correspond to in‐plane defects whose radiative efficiency is strongly influenced by their local environment. In this picture, annealing above 500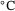 may influence the SEM‐induced carbonaceous layer and/or other surface adsorbates, thereby modifying the local dielectric and charge environment. This can enhance non‐radiative decay channels, resulting in a reduction in emission intensity and lifetime. The emitters exhibit a dominant PSB compared to ZPL at RT, emphasizing strong coupling to their surroundings. Additionally, the emitters are located several layers beneath the surface, suggesting that they cannot be attributed purely to surface defects. Taken together, these observations argue against a purely contamination‐based origin of the emitters. Instead, the results support a model in which intrinsic or lattice‐bound defects are responsible for the emission, while their optical activity is strongly modulated by the local environment.

## 3 Conclusion

In summary, a combination of experiments reveals several key characteristics of the green–yellow emitters in hBN that provide insight into their microscopic origin. The emitters are densely generated in regions exposed to electron‐beam irradiation. Their creation is demonstrated even in flakes as thin as 6.4 nm, and stable emission from SPE is still observed after further thinning to 5.2 nm, highlighting their compatibility with nanoscale and near‐field photonic platforms where reduced thickness is advantageous. SEM and AFM imaging indicate local modification in the irradiated regions, indicating electron‐beam‐induced deposition of a carbonaceous layer related to hydrocarbon contamination. Hyperspectral mapping further shows a strong spatial correlation between these modified regions and the emitter distribution, suggesting a possible role of such residues or carbon‐related species in the formation process. At RT, the emission is dominated by the PSB, which indicates a strong coupling to the environment. Annealing experiments provide additional insight into their stability and environmental sensitivity. The emitters exhibit stable spectral signatures without blinking or bleaching up to 500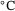, indicating a robust underlying defect structure. The pronounced sensitivity of the emission intensity and lifetime to thermal treatment highlights the strong coupling between these emitters and their local environment. This environmental dependence suggests potential for sensing applications in integrated photonic systems where local heating may play a role. Overall, the experimental evidence rules out a purely surface adsorbates/organic molecular contamination‐based origin for the observed green–yellow quantum emitters in hBN. However, the possibility of interstitial contributions, or a dependence on the SEM‐induced carbonaceous deposition, cannot be fully excluded and needs further investigation. Future experiments could help disentangle these effects, for example, by applying in‐plane and out‐of‐plane electric fields to probe dipole orientation [19, 63, 64] and/or by systematically controlling and reducing hydrocarbon exposure in the SEM chamber [52] to directly assess its role in emitter activation and quenching.

## 4 Experimental Section

### 4.1 Emitter Creation

All the emitters in this work were created by electron irradiation in exfoliated hBN flakes, as described in previous work [50]. A specific area was defined in the software and filled with electron beam irradiation with 7.7 × 10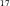 ${\mathrm{cm}}^{-2}$ fluence, 3 kV acceleration voltage, 25 pA current, and 10 seconds dwell time. No pre‐ or post‐treatment was required.

### 4.2 Sample Treatment

To investigate the thermal stability of the emitters, annealing was performed using a tube furnace system (AccuThermo AW‐610). Prior to heating, the chamber was purged with nitrogen ($N_{2}$) to remove residual oxygen. The samples were then annealed under a flowing argon (Ar) atmosphere at the target temperature for specified durations of 30 min. After annealing, the samples were allowed to cool down to RT under Ar environment.

For the vertical localization experiment, the hBN layers were etched using a low‐power RF oxygen plasma system (SPI Plasma Prep plasma etcher/cleaner, Structure Probe, Inc.). The etching was carried out in multiple sequential rounds. In the first three rounds, the sample was exposed to plasma for 20 s each, at chamber pressures of approximately 575, 620, and 700 mTorr, respectively. For all etching steps, the RF power was maintained at 100 W.

### 4.3 Optical Characterization

Hyperspectral imaging measurements were performed using a closed‐loop cryostat (attoDry 800, attocube systems AG). The sample was excited with a 515 nm diode laser (Roithner Lasertechnik GmbH). The excitation beam was passed through a linear polarizer followed by a quarter‐wave plate to generate circularly polarized light, ensuring uniform excitation of emitters regardless of their dipole orientation. The beam was then focused onto the sample using a low‐temperature compatible objective with an NA of 0.82 (attocube systems AG). The PL signal from the sample was collected through the same objective in a confocal configuration. The collected emission was spectrally filtered using a 532 nm long‐pass filter to suppress the excitation laser and subsequently directed to a spectrometer (Teledyne Princeton Instruments) for spectral acquisition.

For temperature‐dependent measurements, the sample temperature was controlled by varying the current through a 100 Ω carbon resistor placed below the sample. Temperature calibration was performed using a 1 kΩ Allen‐Bradley carbon resistor.

Photophysical characterization following oxygen plasma treatment and annealing was performed at RT. At RT, the analysis was based on the PSB because the green‐yellow emitters exhibit a well‐resolved PSB while maintaining single‐photon purity [34, 50]. The emitters were excited using a 532 nm, 80 MHz laser coupled to a commercially available confocal PL setup (MicroTime 200, PicoQuant). The 532 nm excitation wavelength was selected based on our previous finding that excitation through the PSB results in a significantly enhanced emission intensity [14]. The excitation beam was circularly polarized to ensure uniform excitation of emitters with arbitrary dipole orientations. The emitted PL was separated from the excitation path using a 550 nm dichroic mirror. For measurements following oxygen plasma treatment, an additional 550 nm long‐pass filter was used in the detection path. For annealed samples, a 532 nm long‐pass filter was employed to allow broader spectra collection. Due to the fixed 550 nm dichroic mirror in the optical path, spectral information below 550 nm is attenuated and therefore not considered reliable in the analysis.

## Notes

We acknowledge the use of AI assistance to improve the Python script that plotted the figures and rephrased parts of the manuscript for readability. All scientific interpretations, data analyses, and final writing decisions were made by the authors. The authors declare no competing financial interest.

## Conflicts of Interest

The authors declare no conflicts of interest.

## Supporting information


**Supporting File 1**: advs77322‐sup‐0001‐SuppMat.gif.

**Supporting File 2**: advs77322‐sup‐0002‐SuppMat.pdf.

## Data Availability

The data that support the findings of this study are available from the corresponding author upon reasonable request.

## References

[1] G. Cassabois , P. Valvin , and B. Gil , “Hexagonal Boron Nitride Is an Indirect Bandgap Semiconductor,” Nature Photonics 10, no. 4 (2016): 262–266.

[2] B. Shevitski , S. M. Gilbert , C. T. Chen , et al., “Blue‐Light‐Emitting Color Centers in High‐Quality Hexagonal Boron Nitride,” Physical Review B 100, no. 15 (2019): 155419.

[3] C. Cholsuk , S. Suwanna , and T. Vogl , “Tailoring the Emission Wavelength of Color Centers in Hexagonal Boron Nitride for Quantum Applications,” Nanomaterials 12, no. 14 (2022): 2427. 35889651 10.3390/nano12142427 PMC9323195

[4] C. Cholsuk , A. Zand , A. Çakan , and T. Vogl , “The hBN Defects Database: A Theoretical Compilation of Color Centers in Hexagonal Boron Nitride,” Journal of Physical Chemistry C 128, no. 30 (2024): 12716–12725.

[5] R. Camphausen , L. Marini , S. A. Tawfik , T. T. Tran , M. J. Ford , and S. Palomba , “Observation of Near‐Infrared Sub‐Poissonian Photon Emission in Hexagonal Boron Nitride at Room Temperature,” APL Photonics 5, no. 7 (2020): 076103.

[6] R. Bourrellier , S. Meuret , A. Tararan , et al., “Bright UV Single‐Photon Emission at Point Defects in h‐BN,” Nano Letters 16, no. 7 (2016): 4317–4321. 27299915 10.1021/acs.nanolett.6b01368

[7] C. Li , J. E. Froch , M. Nonahal , et al., “Integration of hBN Quantum Emitters in Monolithically Fabricated Waveguides,” ACS Photonics 8, no. 10 (2021): 2966–2972.

[8] A. Zalogina , N. Coste , C. Chen , J. Kim , and I. Aharonovich , “Engineering Quantum Light: Emitters, Photonic Structures, and On‐Chip Integration,” Laser & Photonics Reviews 20, no. 5 (2026): e02309.

[9] M. N. Mishuk , M. Hazra , A. Kumar , P. Dannberg , A. Çakan , and T. Vogl , “All‐Dry Pick‐Up and Transfer Method for Quantum Emitter Arrays in Hexagonal Boron Nitride,” APL Photonics 10, no. 12 (2025): 126107.

[10] D. Gérard , M. Rosticher , K. Watanabe , et al., “Top‐Down Integration of an hBN Quantum Emitter in a Monolithic Photonic Waveguide,” Applied Physics Letters 122, no. 26 (2023): 264001.

[11] J. E. Fröch , L. P. Spencer , M. Kianinia , et al., “Coupling Spin Defects in Hexagonal Boron Nitride to Monolithic Bullseye Cavities,” Nano Letters 21, no. 15 (2021): 6549–6555. 34288695 10.1021/acs.nanolett.1c01843

[12] D. Gérard , A. Pierret , H. Fartas , et al., “Quantum Efficiency and Vertical Position of Quantum Emitters in hBN Determined by Purcell Effect in Hybrid Metal‐Dielectric Planar Photonic Structures,” ACS Photonics 11, no. 12 (2024): 5188–5194.

[13] T. Vogl , H. Knopf , M. Weissflog , P. K. Lam , and F. Eilenberger , “Sensitive Single‐Photon Test of Extended Quantum Theory with Two‐Dimensional Hexagonal Boron Nitride,” Physical Review Research 3, no. 1 (2021): 013296.

[14] M. Hazra , M. Rieger , A. Kumar , et al., “Temperature‐Dependent Emission Spectroscopy of Quantum Emitters in Hexagonal Boron Nitride,” ACS Photonics 13, no. 4 (2026): 1176–1184.

[15] H. Akbari , W.‐H. Lin , B. Vest , P. K. Jha , and H. A. Atwater , “Temperature‐Dependent Spectral Emission of Hexagonal Boron Nitride Quantum Emitters on Conductive and Dielectric Substrates,” Physical Review Applied 15, no. 1 (2021): 014036.

[16] O. Arı , N. Polat , V. Fırat , O. Çakır , and S. Ates , “Temperature‐Dependent Spectral Properties of Hexagonal Boron Nitride Color Centers,” ACS Photonics 12, no. 3 (2025): 1676–1682.

[17] A. Gottscholl , M. Diez , V. Soltamov , et al., “Spin Defects in hBN as Promising Temperature, Pressure, and Magnetic Field Quantum Sensors,” Nature Communications 12, no. 1 (2021): 4480. 10.1038/s41467-021-24725-1 PMC8298442 34294695

[18] G. Grosso , H. Moon , B. Lienhard , et al., “Tunable and High‐Purity Room‐Temperature Single‐Photon Emission from Atomic Defects in Hexagonal Boron Nitride,” Nature Communications 8, no. 1 (2017): 1–8. 10.1038/s41467-017-00810-2 PMC5615041 28951591

[19] I. Zhigulin , J. Horder , V. Ivády , et al., “Stark Effect of Blue Quantum Emitters in Hexagonal Boron Nitride,” Physical Review Applied 19, no. 4 (2023): 044011.

[20] H. L. Stern , Q. Gu , J. Jarman , et al., “Room‐Temperature Optically Detected Magnetic Resonance of Single Defects in Hexagonal Boron Nitride,” Nature Communications 13, no. 1 (2022): 618. 10.1038/s41467-022-28169-z PMC8807746 35105864

[21] Z. Mu , H. Cai , D. Chen , et al., “Excited‐State Optically Detected Magnetic Resonance of Spin Defects in Hexagonal Boron Nitride,” Physical Review Letters 128, no. 21 (2022): 216402. 35687466 10.1103/PhysRevLett.128.216402

[22] Ç. Samaner , S. Paçal , G. Mutlu , K. Uyanık , and S. Ateş , “Free‐Space Quantum Key Distribution with Single Photons from Defects in Hexagonal Boron Nitride,” Advanced Quantum Technologies 5, no. 9 (2022): 2200059.

[23] L. O. Conlon , T. Vogl , C. D. Marciniak , et al., “Approaching Optimal Entangling Collective Measurements on Quantum Computing Platforms,” Nature Physics 19, no. 3 (2023): 351–357. 36942094 10.1038/s41567-022-01875-7 PMC10020085

[24] M. Hoese , M. K. Koch , F. Breuning , N. Lettner , K. G. Fehler , and A. Kubanek , “Single‐Photon Randomness Originating from the Symmetric Dipole Emission Pattern of Quantum Emitters,” Applied Physics Letters 120, no. 4 (2022): 044001.

[25] N. Ahmadi , S. Schwertfeger , P. Werner , et al., “ ${\mathrm{Quick}}^{3}$‐Design of a Satellite‐Based Quantum Light Source for Quantum Communication and Extended Physical Theory Tests in Space,” Advanced Quantum Technologies 7, no. 4 (2024): 2300343.

[26] T. Vogl , K. Sripathy , A. Sharma , et al., “Radiation Tolerance of Two‐Dimensional Material‐Based Devices for Space Applications,” Nature Communications 10, no. 1 (2019): 1202. 10.1038/s41467-019-09219-5 PMC6416293 30867428

[27] T. T. Tran , C. Elbadawi , D. Totonjian , et al., “Robust Multicolor Single‐Photon Emission from Point Defects in Hexagonal Boron Nitride,” ACS Nano 10, no. 8 (2016): 7331–7338. 27399936 10.1021/acsnano.6b03602

[28] Z. Shotan , H. Jayakumar , C. R. Considine , et al., “Photoinduced Modification of Single‐Photon Emitters in Hexagonal Boron Nitride,” ACS Photonics 3, no. 12 (2016): 2490–2496.

[29] N. Mendelson , Z.‐Q. Xu , T. T. Tran , et al., “Engineering and Tuning of Quantum Emitters in Few‐Layer Hexagonal Boron Nitride,” ACS Nano 13, no. 3 (2019): 3132–3140. 30715854 10.1021/acsnano.8b08511

[30] L. J. Martinez , T. Pelini , V. Waselowski , et al., “Efficient Single‐Photon Emission from a High‐Purity Hexagonal Boron Nitride Crystal,” Physical Review B 94, no. 12 (2016): 121405.

[31] N. R. Jungwirth and G. D. Fuchs , “Optical Absorption and Emission Mechanisms of Single Defects in Hexagonal Boron Nitride,” Physical Review Letters 119, no. 5 (2017): 057401. 28949753 10.1103/PhysRevLett.119.057401

[32] T. Vogl , Y. Lu , and P. K. Lam , “Room‐Temperature Single‐Photon Source Using Fiber‐Integrated Hexagonal Boron Nitride,” Journal of Physics D: Applied Physics 50, no. 29 (2017): 295101.

[33] T. Vogl , M. W. Doherty , B. C. Buchler , Y. Lu , and P. K. Lam , “Atomic Localization of Quantum Emitters in Multilayer Hexagonal Boron Nitride,” Nanoscale 11, no. 30 (2019): 14362–14371. 31332410 10.1039/c9nr04269e

[34] A. Kumar , C. Samaner , C. Cholsuk , et al., “Polarization Dynamics of Solid‐State Quantum Emitters,” ACS Nano 18, no. 7 (2024): 5270–5281. 38335970 10.1021/acsnano.3c08940 PMC10883057

[35] T. T. Tran , K. Bray , M. J. Ford , M. Toth , and I. Aharonovich , “Quantum Emission from Hexagonal Boron Nitride Monolayers,” Nature Nanotechnology 11, no. 1 (2016): 37–41. 10.1038/nnano.2015.242 26501751

[36] A. L. Exarhos , D. A. Hopper , R. R. Grote , A. Alkauskas , and L. C. Bassett , “Optical Signatures of Quantum Emitters in Suspended Hexagonal Boron Nitride,” ACS Nano 11, no. 3 (2017): 3328–3336. 28267917 10.1021/acsnano.7b00665

[37] A. W. Schell , M. Svedendahl , and R. Quidant , “Quantum Emitters in Hexagonal Boron Nitride Have Spectrally Tunable Quantum Efficiency,” Advanced Materials 30, no. 14 (2018): 1704237. 10.1002/adma.201704237 29473231

[38] I. Aharonovich , D. Englund , and M. Toth , “Solid‐State Single‐Photon Emitters,” Nature Photonics 10, no. 10 (2016): 631–641.

[39] X. Du , C. Frye , J. Edgar , J. Lin , and H. Jiang , “Temperature Dependence of the Energy Bandgap of Two‐Dimensional Hexagonal Boron Nitride Probed by Excitonic Photoluminescence,” Journal of Applied Physics 115, no. 5 (2014): 053503.

[40] A. B. Dhu‐al Shaik , P. Palla , and D. Jenkins , “Electrical Tuning of Quantum Light Emitters in hBN for Free‐Space and Telecom Optical Bands,” Scientific Reports 14, no. 1 (2024): 811. 38191916 10.1038/s41598-024-51504-x PMC10774371

[41] A. Gottscholl , M. Diez , V. Soltamov , et al., “Room‐Temperature Coherent Control of Spin Defects in Hexagonal Boron Nitride,” Science Advances 7, no. 14 (2021): eabf3630. 33811078 10.1126/sciadv.abf3630 PMC11059373

[42] K. Yamamura , N. Coste , H. Z. J. Zeng , M. Toth , M. Kianinia , and I. Aharonovich , “Quantum Efficiency of the B‐Center in Hexagonal Boron Nitride,” Nanophotonics 14, no. 11 (2025): 1715–1720. 40470093 10.1515/nanoph-2024-0412 PMC12133246

[43] M. Neumann , X. Wei , L. Morales‐Inostroza , et al., “Organic Molecules as Origin of Visible‐Range Single‐Photon Emission from Hexagonal Boron Nitride and Mica,” ACS Nano 17, no. 12 (2023): 11679–11691. 37276077 10.1021/acsnano.3c02348

[44] A. Neumann , J. Lindlau , S. Thoms , T. Basché , and A. Högele , “Accidental Contamination of Substrates and Polymer Films by Organic Quantum Emitters,” Nano Letters 19, no. 5 (2019): 3207–3213. 30985126 10.1021/acs.nanolett.9b00712 PMC6542549

[45] S. Zhao , J. Lavie , L. Rondin , et al., “Single‐Photon Emission from Graphene Quantum Dots at Room Temperature,” Nature Communications 9, no. 1 (2018): 3470. 10.1038/s41467-018-05888-w PMC6110725 30150689

[46] Q. Chen , S. Thoms , S. Stöttinger , et al., “Dibenzo [hi, st] Ovalene as Highly Luminescent Nanographene: Efficient Synthesis via Photochemical Cyclodehydroiodination, Optoelectronic Properties, and Single‐Molecule Spectroscopy,” Journal of the American Chemical Society 141, no. 41 (2019): 16439–16449. 31589425 10.1021/jacs.9b08320

[47] J. Macklin , J. Trautman , T. Harris , and L. Brus , “Imaging and Time‐Resolved Spectroscopy of Single Molecules at an Interface,” Science 272, no. 5259 (1996): 255–258.

[48] F. Qazi , E. Shahsavari , S. Prawer , A. S. Ball , and S. Tomljenovic‐Hanic , “Detection and Identification of Polyaromatic Hydrocarbons (PAHs) Contamination in Soil Using Intrinsic Fluorescence,” Environmental Pollutants 272 (2021): 116010. 10.1016/j.envpol.2020.116010 33189449

[49] A. Shkarin and S. Götzinger , “Organic Molecules as Single‐Photon Sources,” Applied Physics Reviews 13, no. 2 (2026): 021312.

[50] A. Kumar , C. Cholsuk , A. Zand , et al., “Localized Creation of Yellow Single‐Photon‐Emitting Carbon Complexes in Hexagonal Boron Nitride,” APL Materials 11, no. 7 (2023): 071108.

[51] M. T. Postek , “An Approach to the Reduction of Hydrocarbon Contamination in the Scanning Electron Microscope,” Scanning 18, no. 4 (1996): 269–274.

[52] S. Nedić , K. Yamamura , A. Gale , I. Aharonovich , and M. Toth , “Electron Beam Restructuring of Quantum Emitters in Hexagonal Boron Nitride,” Advanced Optical Materials 12, no. 24 (2024): 2400908.

[53] T. Vogl , G. Campbell , B. C. Buchler , Y. Lu , and P. K. Lam , “Fabrication and Deterministic Transfer of High‐Quality Quantum Emitters in Hexagonal Boron Nitride,” ACS Photonics 5, no. 6 (2018): 2305–2312.

[54] Y. Chen , A. Gale , K. Yamamura , et al., “Annealing of Blue Quantum Emitters in Carbon‐Doped Hexagonal Boron Nitride,” Applied Physics Letters 123, no. 4 (2023): 041902.

[55] K. P. O'donnell and X. Chen , “Temperature Dependence of Semiconductor Band Gaps,” Applied Physics Letters 58, no. 25 (1991): 2924–2926.

[56] Q. Tan , J.‐M. Lai , X.‐L. Liu , et al., “Donor–Acceptor Pair Quantum Emitters in Hexagonal Boron Nitride,” Nano Letters 22, no. 3 (2022): 1331–1337. 35073101 10.1021/acs.nanolett.1c04647

[57] M. Fischer , A. Sajid , J. Iles‐Smith , et al., “Combining Experiments on Luminescent Centres in Hexagonal Boron Nitride with the Polaron Model and Ab Initio Methods towards the Identification of Their Microscopic Origin,” Nanoscale 15, no. 34 (2023): 14215–14226. 37594441 10.1039/d3nr01511d PMC10472209

[58] L. Liu , I. Khanonkin , J. Eberle , et al., “Single‐Photon Emitters in Ultra‐Thin Hexagonal Boron Nitride Layers,”*arXiv preprint arXiv:2507.02633* (2025).

[59] A. Kumar , C. Cholsuk , M. N. Mishuk , et al., “Comparative Study of Quantum Emitter Fabrication in Wide Bandgap Materials Using Localized Electron Irradiation,” ACS Applied Optical Materials 2, no. 2 (2024): 323–332.

[60] A. Zobelli , C. Ewels , A. Gloter , and G. Seifert , “Vacancy Migration in Hexagonal Boron Nitride,” Physical Review B 75, no. 9 (2007): 094104.

[61] J. Serrano , A. Bosak , R. Arenal , et al., “Vibrational Properties of Hexagonal Boron Nitride: Inelastic X‐Ray Scattering and Ab Initio Calculations,” Physical Review Letters 98, no. 9 (2007): 095503. 17359168 10.1103/PhysRevLett.98.095503

[62] M. Hoese , P. Reddy , A. Dietrich , et al., “Mechanical Decoupling of Quantum Emitters in Hexagonal Boron Nitride from Low‐Energy Phonon Modes,” Science Advances 6, no. 40 (2020): eaba6038. 32998895 10.1126/sciadv.aba6038 PMC7527221

[63] N. Nikolay , N. Mendelson , N. Sadzak , et al., “Very Large and Reversible Stark‐Shift Tuning of Single Emitters in Layered Hexagonal Boron Nitride,” Physical Review Applied 11, no. 4 (2019): 041001.

[64] G. Noh , D. Choi , J.‐H. Kim , et al., “Stark Tuning of Single‐Photon Emitters in Hexagonal Boron Nitride,” Nano Letters 18, no. 8 (2018): 4710–4715. 29932664 10.1021/acs.nanolett.8b01030

