# Supplementary material for: Insights Into the Nature of Quantum Emitters in Electron‐Irradiated Hexagonal Boron Nitride
Source: Adv Sci (Weinh). 2026 Aug 31:e77322. Online ahead of print. doi: 10.1002/advs.77322 (PMC13527696; doi:10.1002/advs.77322)
Supplement: Supplementary file 1 [file ADVS-9999-e77322-s002.pdf]

Supplementary Information:

Insights into the Nature of Quantum Emitters in Electron-Irradiated Hexagonal Boron Nitride

Mouli Hazra[1,3,*] *Anna Rupp*[2] *Mohammad N. Mishuk*[1,3] *Josefine Krause*[1,3,6] *Anand Kumar*[1,3] *Julien Chénedé*[1,3] *Mingi Kang*[1,3] *Bayarjargal N. Tugchin*[4] *Marijn Rikers*[4,7,8] *Isabelle Staude*[4,7] *Thomas Pertsch*[4,5,6] *Alexander Högele*[2,3] *Tobias Vogl*[1,3,*]

[1] Department of Computer Engineering, TUM School of Computation, Information and Technology, Technical University of Munich, 80333 Munich, Germany
[2] Fakultät für Physik, Munich Quantum Center, and Center for NanoScience (CeNS), Ludwig-Maximilians-Universität München, 80539 Munich, Germany
[3] Munich Center for Quantum Science and Technology (MCQST), 80799 Munich, Germany
[4] Institute of Applied Physics, Abbe Center of Photonics, Friedrich Schiller University Jena, Albert-Einstein-Straße 6, 07745 Jena, Germany
[5] Fraunhofer-Institute for Applied Optics and Precision Engineering IOF, Albert-Einstein-Str. 7, 07745 Jena, Germany
[6] Max Planck School of Photonics, Hans-Knöll-Str. 1, 07745 Jena, Germany
[7] Institute of Solid State Physics, Friedrich Schiller University Jena, Max-Wien-Platz 1, 07743 Jena, Germany
[8] ARC Centre of Excellence for Transformative Meta-Optical Systems, Department of Quantum Science and Technology, Research School of Physics, Australian National University, Canberra, ACT 2601, Australia

[*] Corresponding author: `mouli.hazra@tum.de; tobias.vogl@tum.de`

1 Correlation Between Electron Beam Irradiation and Green–Yellow Emitter Formation

In this work, two types of electron-beam-irradiated structures were investigated: (i) small dot-like regions with a diameter of $\simeq 300$ nm, where the electron beam was focused on a single spot for 10 s, and (ii) few-micron-wide circular or square regions, where an area was defined and uniformly scanned using the same irradiation parameters. Atomic force microscopy (AFM) in Figure. **S1** reveals a clear height difference between the pristine and irradiated regions. The irradiated areas exhibit an average height increase of approximately 2 nm relative to the surrounding pristine hBN. This increase is not spatially uniform, with occasional hills and dips observed within the irradiated regions.

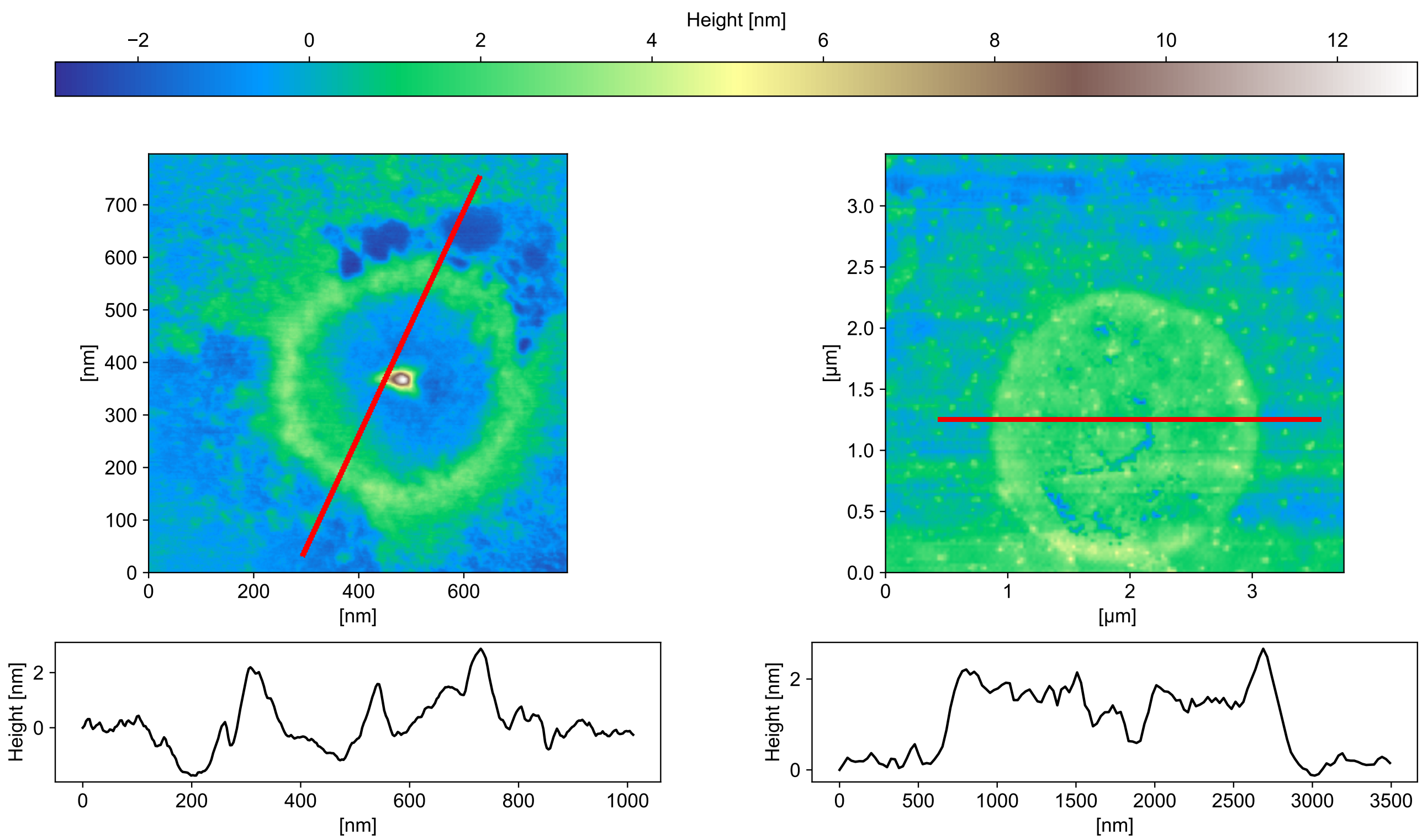

Figure S1: AFM height maps of an electron irradiated hBN flake showing a small irradiated dot (left) and a large irradiated area (right), highlighting the height changes in the irradiated region with respect to the pristine area. The height profile along the red lines is shown in the bottom panel for the respective columns.

The spatial distribution of emitters around such irradiated regions is shown in Figure. **S2** (a-c) using PL lifetime mapping. The blue regions correspond to the electron-irradiated areas, while the red spots indicate the locations of the green–yellow emitters. These emitters exhibit characteristic lifetimes of approximately 3–5 ns. This assignment is supported by our previous comprehensive characterization of these emitters across multiple samples, as detailed in the Supplementary Information of Ref. [1]. Based on hyperspectral PL mapping, emitters with other spectral emissions are not observed around the irradiated regions. Therefore, diffraction-limited spots with lifetimes in the 3–5 ns range can be reliably attributed to green–yellow emitters. For the dot-like irradiated structures, green-yellow emitters are typically observed adjacent to (< 1 μm) or at the edges of the irradiated spots. However, because the irradiated dots have a diameter of approximately 300 nm [2] (comparable to the optical diffraction

limit), the exact emitter positions cannot be determined with higher precision. In most cases, one or two emitters are detected around each irradiated dot, although some dots do not produce any observable emitters [2, 1]. In contrast, the larger irradiated regions exhibit a much higher density of green-yellow emitters. These emitters are predominantly located directly on the irradiated area, with additional emitters found up to approximately < 1 μm beyond the irradiated boundary. We have selected such large irradiated structures for hyperspectral imaging, as they provide a sufficiently high emitter density for meaningful statistical analysis. They also facilitate post-annealing studies because the irradiated regions remain easily identifiable even after additional emitters are generated uniformly across the previously pristine areas during annealing. Overall, our investigations across multiple samples over several years, together with our previous studies [2, 1], consistently demonstrate that the formation of emitters is strongly correlated with the electron-irradiated regions.

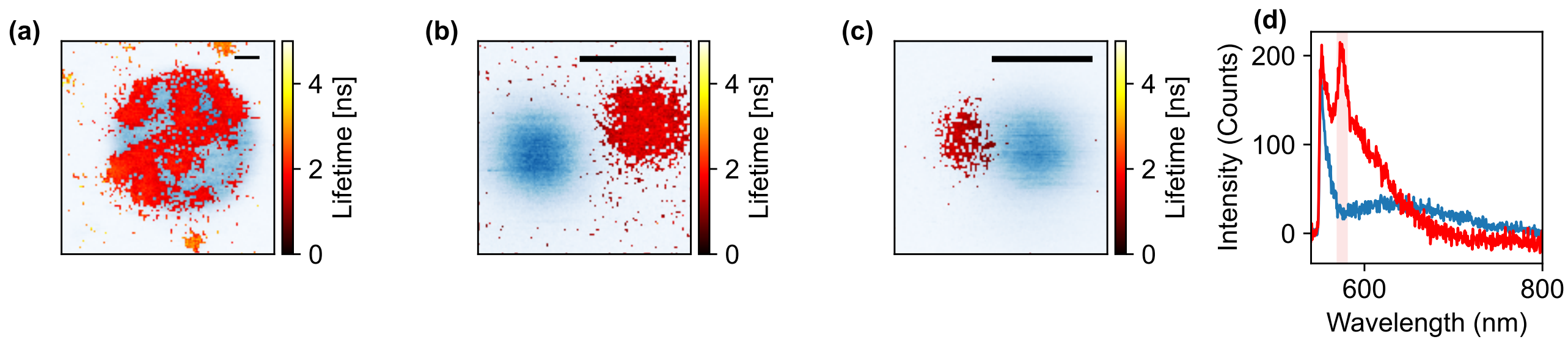

Figure S2: PL lifetime maps showing the spatial distribution of the green-yellow emitters with respect to the irradiated spot. The blue spots are the irradiated regions, and the red spots are where the green-yellow emitters are present. When a few micron-wide areas are irradiated, the emitters are present densely, as in (a) and **Figure. 1(a-c)** in the main text. If an electron beam is hit on a single point for 10 sec, the dot-like regions are created as in (b and c). In that case the emitters are at the edge or slightly away from the irradiated spot. The scale bar is 0.5 μm. The room-temperature PL spectra acquired from the blue (carbonaceous layer) and red (green–yellow emitter) regions are shown in (d). The red-shaded region highlights the PSB of the green–yellow emitter, which is absent in the spectrum acquired from the carbonaceous layer (blue). The spectral region below 560 nm is dominated by residual laser leakage and should therefore be disregarded.

Along with observed topological changes, the evidence of carbon layer deposition on the irradiated region comes from optical measurements. Irradiated regions that do not contain green-yellow emitters exhibit a broad background in their emission spectra, without the characteristic features of the green-yellow emitters as shown in Figure. **S2**(d). This broad emission is consistent with luminescence from the deposited carbonaceous layer [3]. Together with the increase in AFM height within the irradiated region, the characteristic spectral signatures of the carbonaceous layer and the darker contrast observed in the SEM image (Figure. **S3**) provide strong evidence for the formation of a carbonaceous layer in the electron-irradiated region.

It should be noted that the deposition of the carbonaceous layer does not directly correlate with the density of the emitters within the irradiated region. For example, the AFM topography shown in Figure. **S1** (right) and the PL lifetime map in Figure. **S2**(a) correspond to the same electron-irradiated area. Although the irradiated region exhibits a pronounced carbonaceous deposit, the locations of maximum deposit thickness do not coincide with the regions of highest emitter density. This observation indicates that the carbonaceous layer itself is not the green–yellow emitter. Nevertheless, carbon may still play a role in the formation of the emitting defect complex, either as a constituent of the defect or by modifying the local chemical environment during electron irradiation.

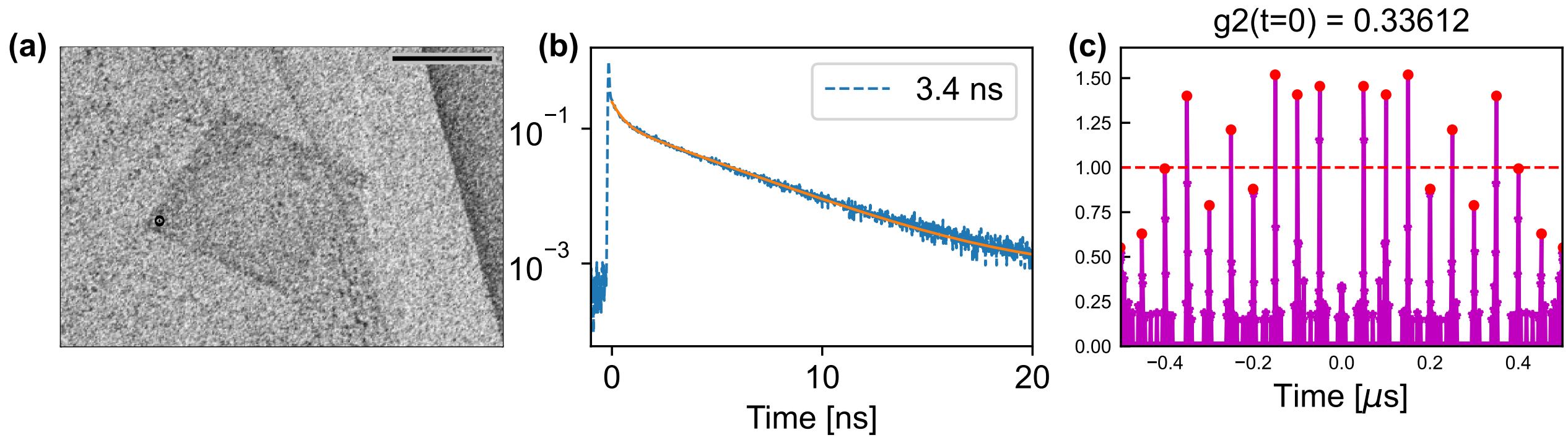

Figure S3: (a) SEM image of the irradiated area shown in **Figure. 1** in main text. The scale bar corresponds to 3 μm. (b,c) Lifetime and $g^{(2)}$ measurements acquired at the position at RT indicated by the black dot in (a).

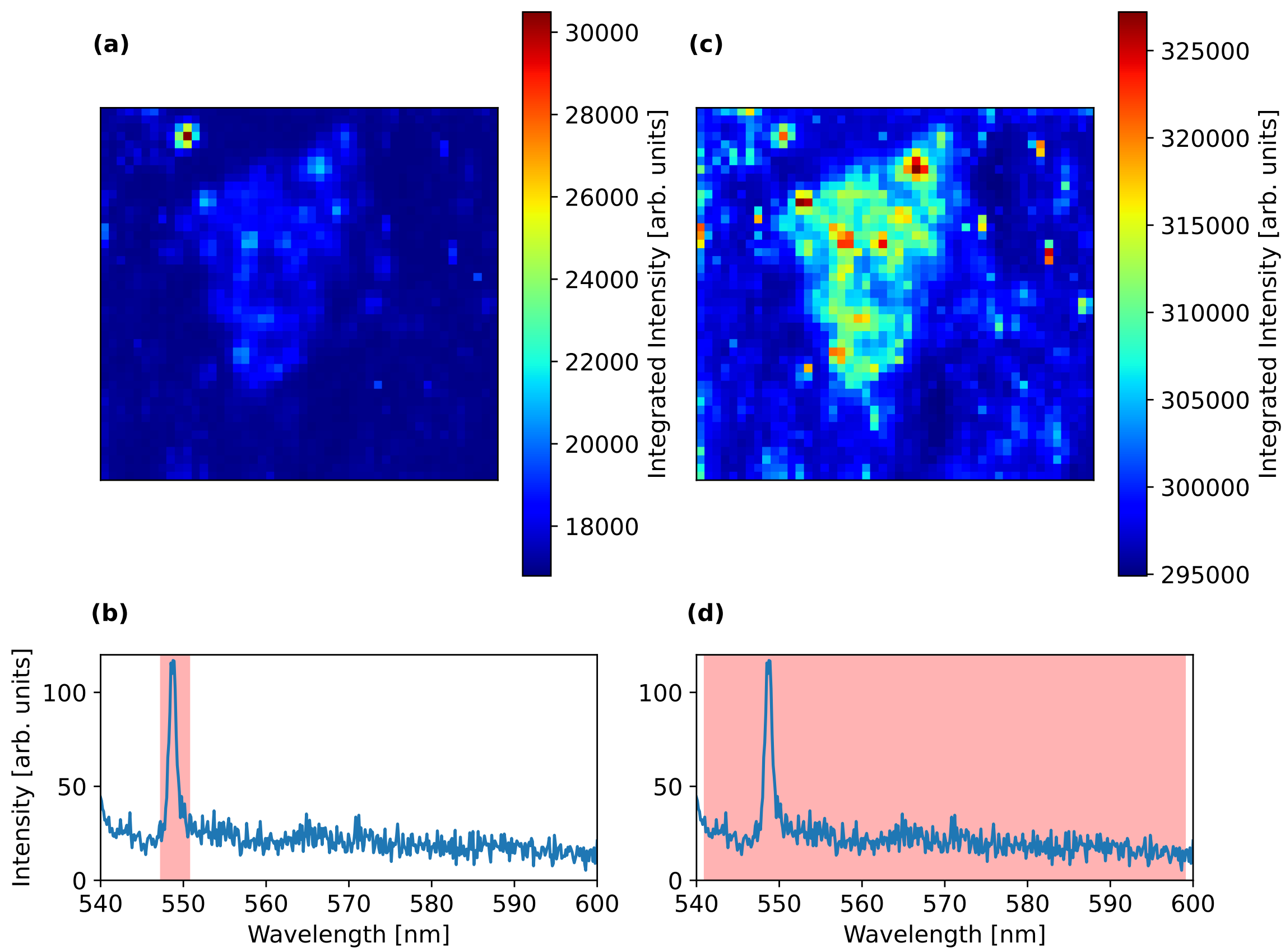

Figure S4: (a) Intensity map at 155 K integrated around the ZPL, showing a diffraction-limited emitter within the irradiated square. (b) Integration over the full spectral range, including background emission likely from hydrocarbons. Typical emitter spectra (c–d), with the integration windows corresponding to panels (a–b) marked in red.

2 Emitter Visibility in Hyperspectral Map

The visibility of individual emitters within a large electron-irradiated region is influenced by the background emission originating from the carbonaceous layer. As shown in Figure. **S4**, integrating the PL intensity over the entire spectral range produces a uniformly bright irradiated region, making many individual emitters difficult to distinguish. In contrast, when

the integration is restricted to the spectral window containing the ZPL, the individual emitters become visible as diffraction-limited circular spots (as seen in the bottom-right corner of the irradiated square). This highlights the advantage of hyperspectral mapping over typical photo-detector scans for PL mapping.

The apparent reduction in emission intensity in the bottom-right corner of the irradiated region is likely associated with the surface morphology of the hBN flake. As visible in the SEM image in Figure. **S3**, the irradiated area is not uniformly flat but contains a fold-like feature. Such morphological inhomogeneities can result in non-uniform carbonaceous deposition and/or may also reduce the optical collection efficiency, leading to a lower observed emission.

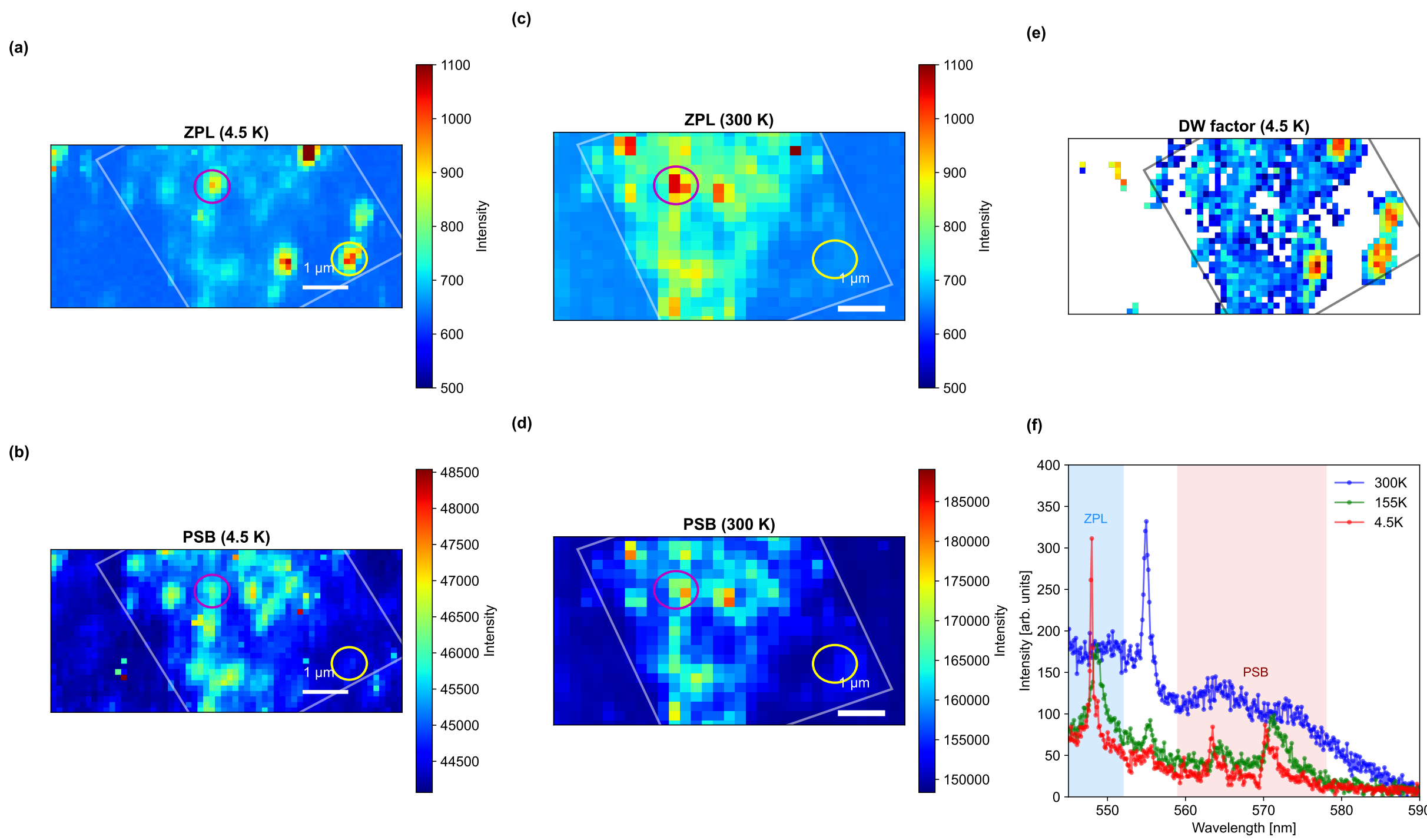

Figure S5: Hyperspectral PL maps obtained by integrating different spectral windows at 4.5 K (a,b) and room temperature (c,d). (e) Two-dimensional map of the Debye–Waller (DW) factor for individual emitters measured at 4.5 K. (f) Representative emission spectrum showing the spectral integration windows used to generate the ZPL maps (a,c) and the PSB maps (b,d).

Electron-phonon coupling of individual emitters can also influence the visibility of the emitter in hyperspectral maps at different temperatures. The low-temperature hyperspectral map reveals that the electron-phonon coupling vary across different emitters. Emitters with a higher DW factor [$\frac{I_{ZPL}}{I_{Total}}$] exhibit dominant ZPL and comparatively weaker PSB. As the temperature increases, thermal broadening reduces the ZPL contrast, while the PSB becomes the dominant spectral feature. This causes the emitters with weaker PSB difficult to detect at RT. In **Figure. 1** of the main text, the hyperspectral maps are generated by integrating the emission intensity over a spectral window centered on the ZPL, thereby demonstrating the temperature-dependent visibility difference between emitters A and B. On the other hand, temperature does not affect the visibility of emitters with greater electron-phonon coupling (i.e., a lower DW factor at 4.5 K). In Figure. **S5** (b,d), hyperspectral maps were plotted with emission spectra integrated over a spectral window centered on the PSB. Disappearance of any emitter from 4.5 K to RT is not evident there.

3 Emitter Classification

The emission spectrum of a typical green-yellow emitter is shown in Figure. **S6**, with the prominent spectral features labeled directly in the figure. The assignment of the individual spectral peaks follows our previous work [4].

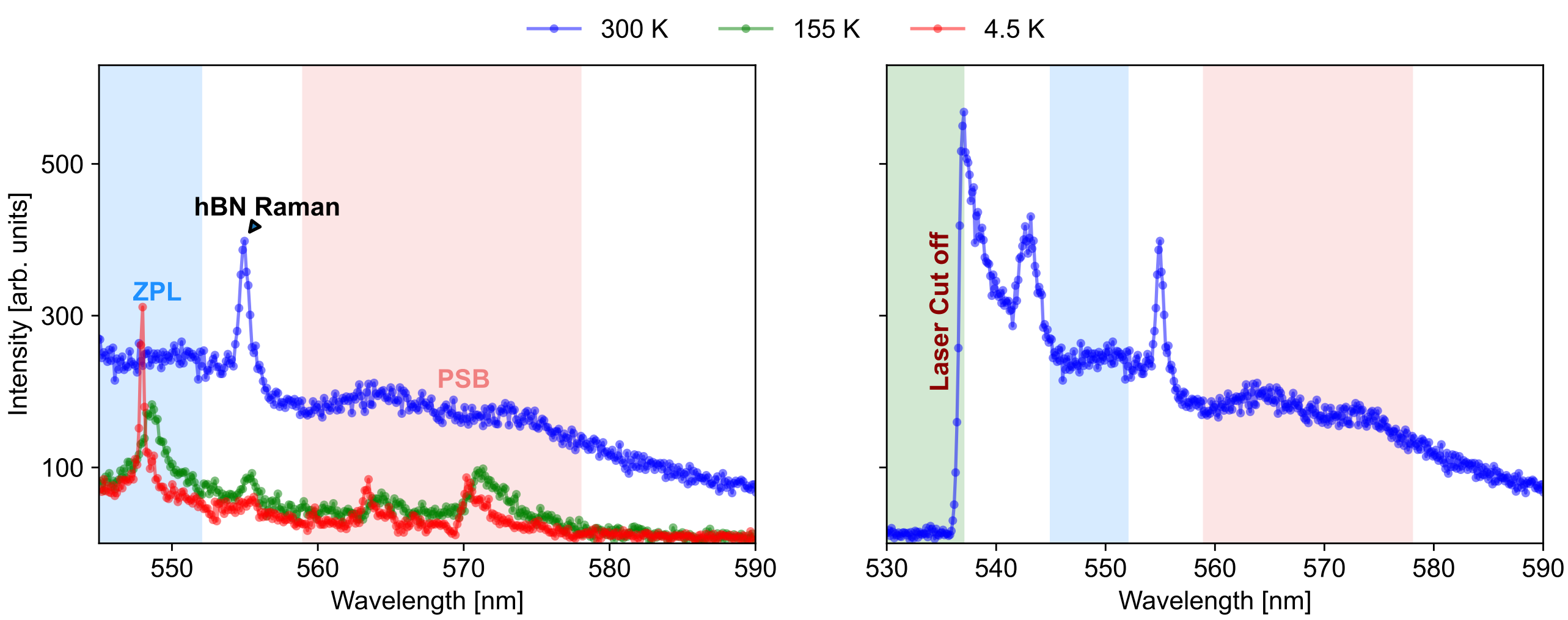

Figure S6: Spectral features of the green-yellow emitter at different temperatures.

At RT, the ZPL loses spectral contrast and, for most emitters, becomes difficult to distinguish from the background as shown in the Figure. **S6**(right). This occurs because the ZPL broadens and decreases in intensity with increasing temperature, eventually becoming difficult to separate from the excitation laser background and the hBN Raman scattering. This hinders reliable fitting of the ZPL across the dataset for meaningful statistical analysis. Therefore, the ZPL at RT is excluded from our analysis in **Figure. 1** in main text and Figure. **S7**. Notably, the emitters retain a high degree of single-photon purity when $g^{(2)}(\tau)$) is measured collecting only the PSB emission at RT, as shown in Figure. **S3**(c). This demonstrates that the PSB originates from the same single-photon emitter and can therefore be used reliably for further characterization at RT. Consequently, in the annealing and vertical localization experiments, all analyses are performed using the PSB emission rather than the broadened ZPL.

Throughout this work, we refer to these green-yellow emitters as belonging to a single class because they are generated by the same electron-irradiation process and exhibit highly consistent emission characteristics. (such as emission spectral and PL lifetime). Our hyperspectral mapping measurements show that the variation in the ZPL energy is limited to within a few nanometers, which is comparable to the spectral diffusion and local environmental variations commonly observed for hBN emitters. We observe slightly more variations in the relative intensity and shape of the PSB, with some emitters exhibiting multiple resolved phonon modes, as shown in **Figure. 1(i)** of the main text. This behavior is expected because the PSB is strongly influenced by the coupling of the emitter to the local crystal environment. Even the PSB variation at RT rarely exceeds 5 nm across all measured emitters. Therefore, the observed spectral differences are consistent with emitter-to-emitter variations arising from local strain, charge fluctuations, or other environmental perturbations, rather than indicating the presence of distinct defect species. On this basis, we consider these emitters to belong to the same class of emitters.

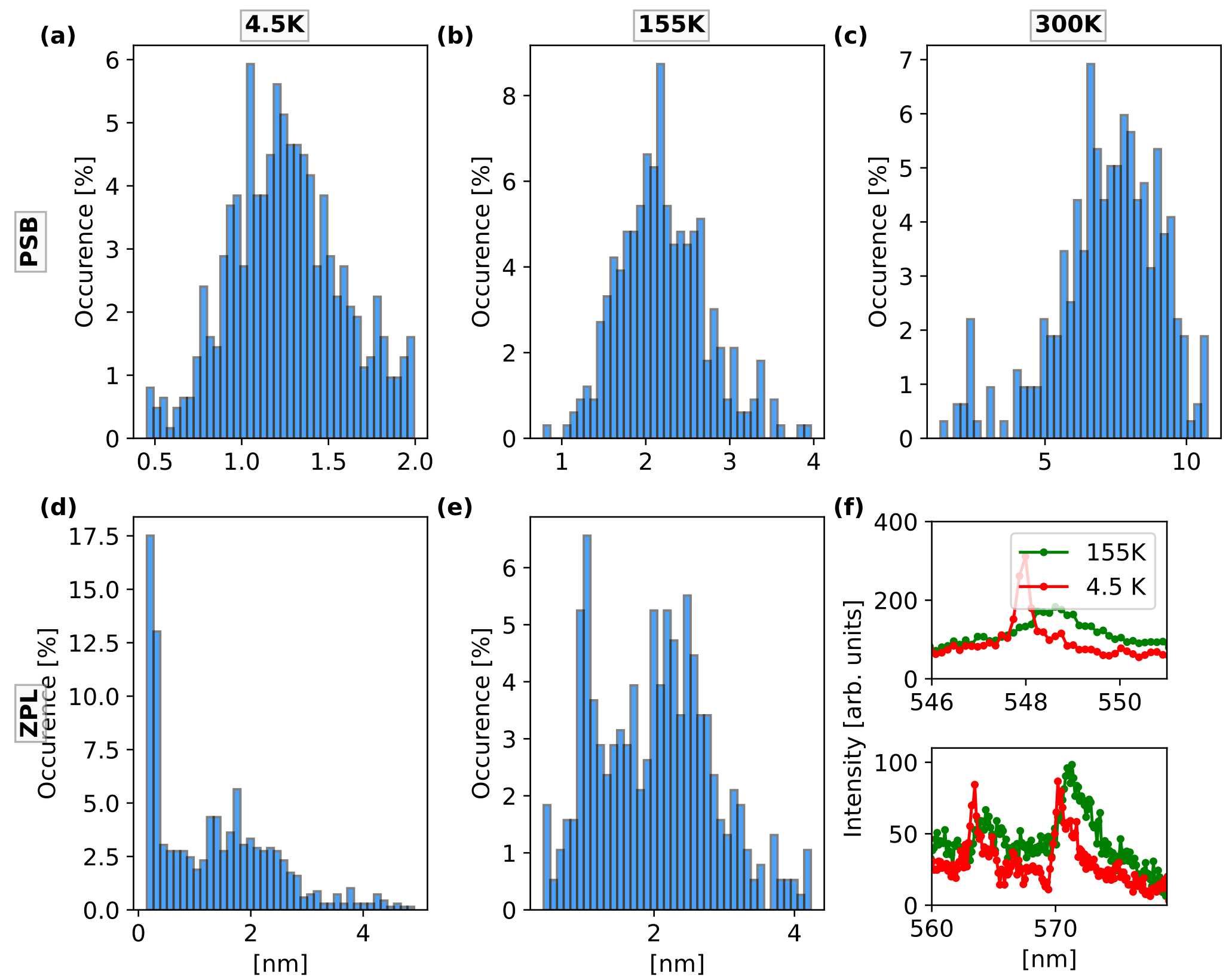

Figure S7: Histogram of the full width at half maximum (FWHM) of the emission peaks within the square irradiated regions shown in **Figure 1** in main text, for the ZPL (a–c) and PSB (d–e). (f) Zoomed-in spectra around the ZPL (top) and PSB (bottom) for point B, as shown in the main text.

4 Annealing-Induced Changes in the Electron-Irradiated hBN

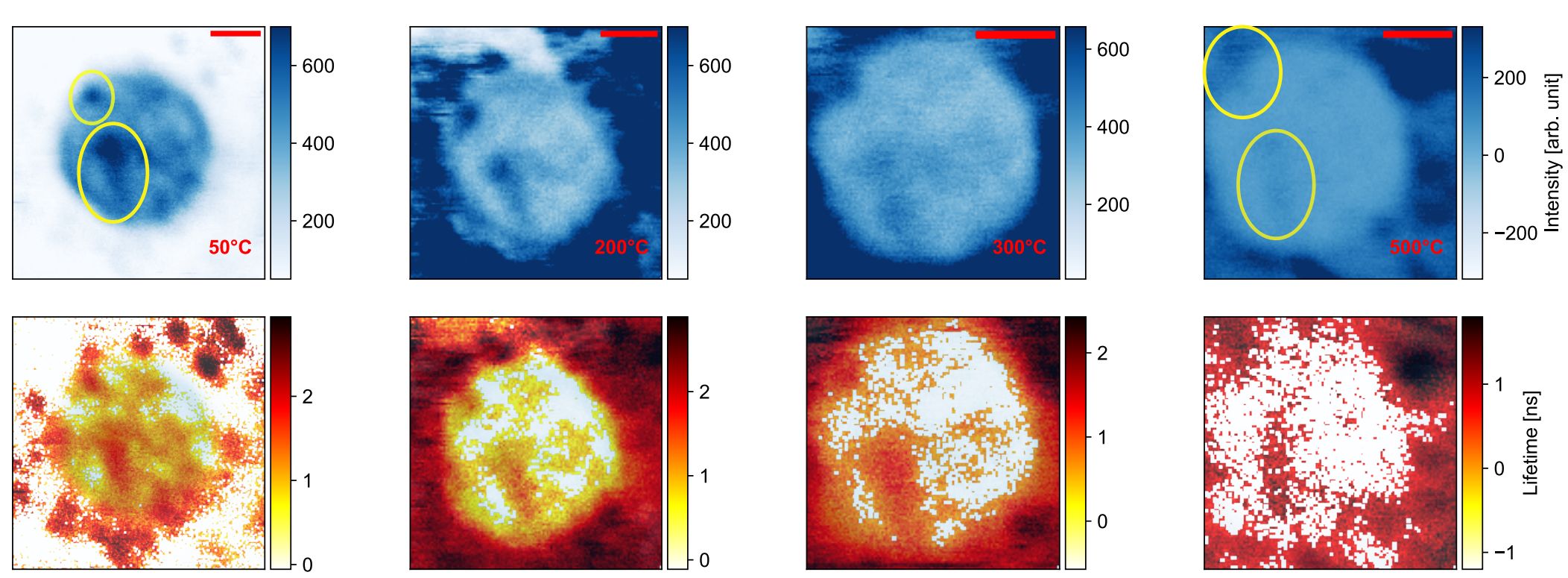

Figure S8: PL intensity (top) and lifetime (bottom) maps of the electron-irradiated region shown in **Figure. 2** of the main text, acquired after annealing at 50°C, 200°C, 300°C, and 500°C temperatures from left to right. The yellow circles mark the representative green-yellow emitters characterized. Although both the PL intensity and the lifetime decrease with increasing annealing temperatures, the emitters remain visible in the maps. Each column uses the same scale bar, corresponding to 1 μm.

The few-micron-wide circular region shown in the annealing experiments in **Figure. 2** of the main text corresponds to the electron-irradiated area. As demonstrated in Figure. **S2**, large-area electron irradiation generates a dense population of green–yellow emitters within the irradiated region. These emitters appear as patches with characteristic PL lifetimes of 3–5 ns in the PL lifetime maps. Before annealing, the irradiated region exhibits a higher PL intensity than the surrounding pristine hBN. However, after annealing, the irradiated region appears darker compared to the pristine area in the PL intensity maps (Figure. **S8**). This is due to the formation of thermally activated emitters. These temporary emitters disappear upon further high-temperature annealing or exposure to high-power laser illumination, as demonstrated in Figure. **S9**. In contrast, although the green–yellow emitters exhibit a gradual decrease in PL intensity with increasing annealing temperature, they remain optically active and continue to emit stably throughout the annealing process, as shown in Figure. **S10**.

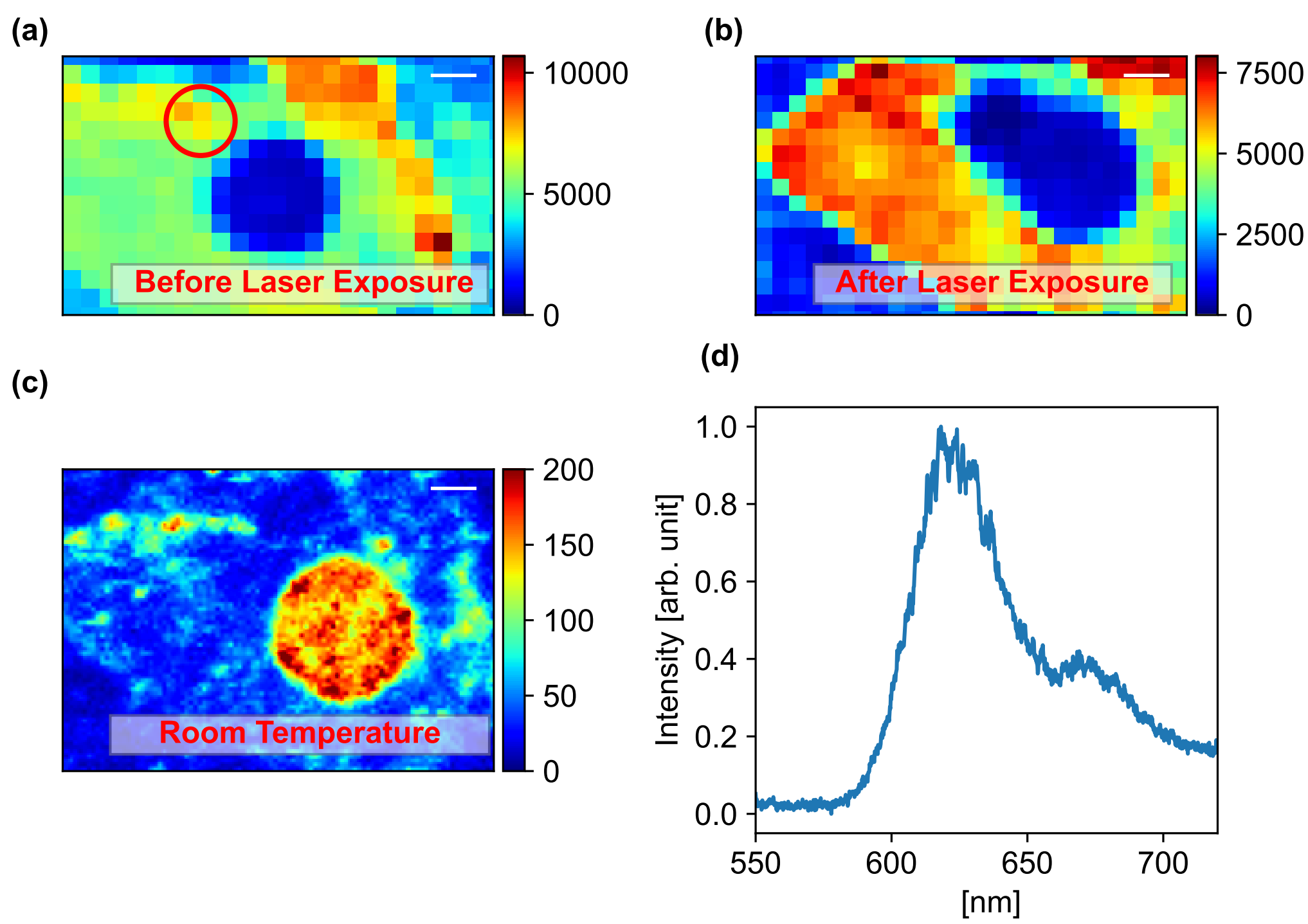

Figure S9: PL intensity maps of the irradiated flake after annealing at 150 °C, shown (a) before and (b) after exposing a selected point to laser exposure. At this temperature, the entire flake exhibits significantly enhanced fluorescence compared to the locally irradiated region. In contrast, at room temperature, the irradiated area appears more fluorescent than the pristine flake. (d) A spectrum acquired from the point indicated by the red circle in (a) does not show the characteristic yellow-emitter peak. Scale bars in (a–c) are 1 µm.

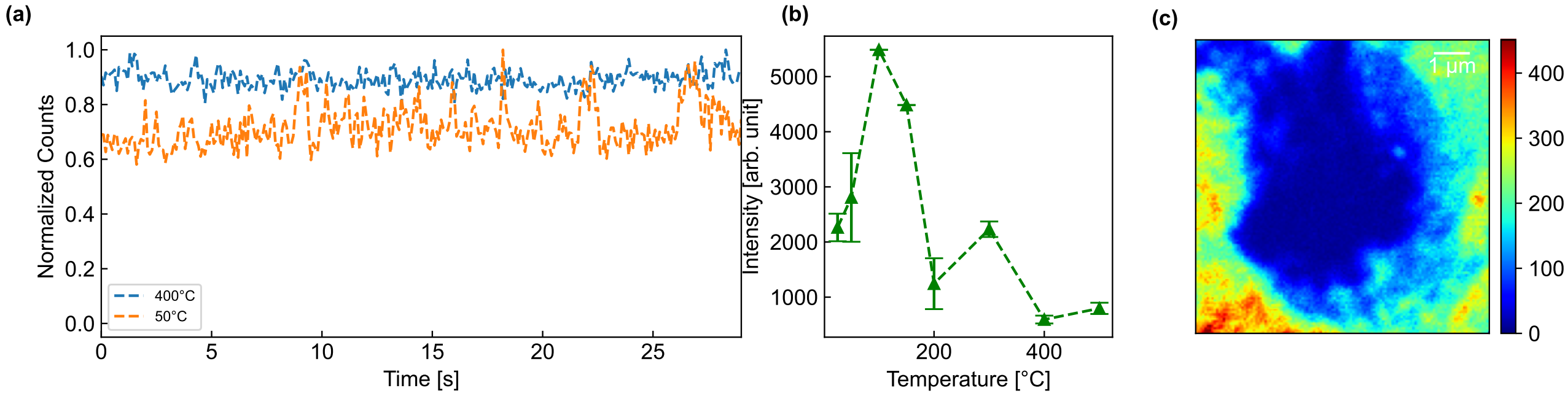

Figure S10: (a) PL intensity time traces recorded at different temperatures for the same emitter shown in **Figure 2(d–f)** in the main text. (b) PSB intensity extracted from Gaussian fits to the spectra at each temperature. While the measured intensity can be influenced by alignment, efforts were made to keep conditions as consistent as possible across temperatures. A clear decrease in intensity is observed with increasing temperature. (c) PL intensity map after annealing at 600 °C for 30 minutes.

5 Probing the Vertical Distribution of Green–Yellow Emitters

This section presents the photophysical characterization of emitters located in different regions of the hBN flake used for the vertical localization experiment shown in **Figure. 3** of the main text.

For the plasma-etching experiments, we primarily relied on PL lifetime mapping to track individual emitters throughout the thinning process. Prior to plasma treatment, the green–yellow emitters were identified and characterized using their emission spectra, PL lifetime, and second-order autocorrelation measurements in both the thicker (Figure. **S11**, **S12**) and thinner regions of the flake (Figure. **S13**). These measurements confirmed the presence of emitters several micrometers away from the irradiated region (Figure. **S11**) as well as in flakes as thin as 6 nm (Figure. **S13**).

Following the initial characterization, subsequent plasma-etching steps were monitored primarily using PL lifetime maps rather than repeating the full photophysical characterization after each etching cycle. Emitters exhibiting the characteristic 3–5 ns lifetime at the same spatial locations were identified as the original green–yellow emitters and used to quantify the surviving emitter population. As shown by the statistics in Figure. **S14**(e), the majority of the initially identified emitters disappear as the hBN flake is progressively thinned. This observation indicates that the emitters are distributed at different depths within the hBN crystal, rather than being confined to the surface or exclusively located at the hBN/substrate interface, as would be expected for contamination-related emitters.

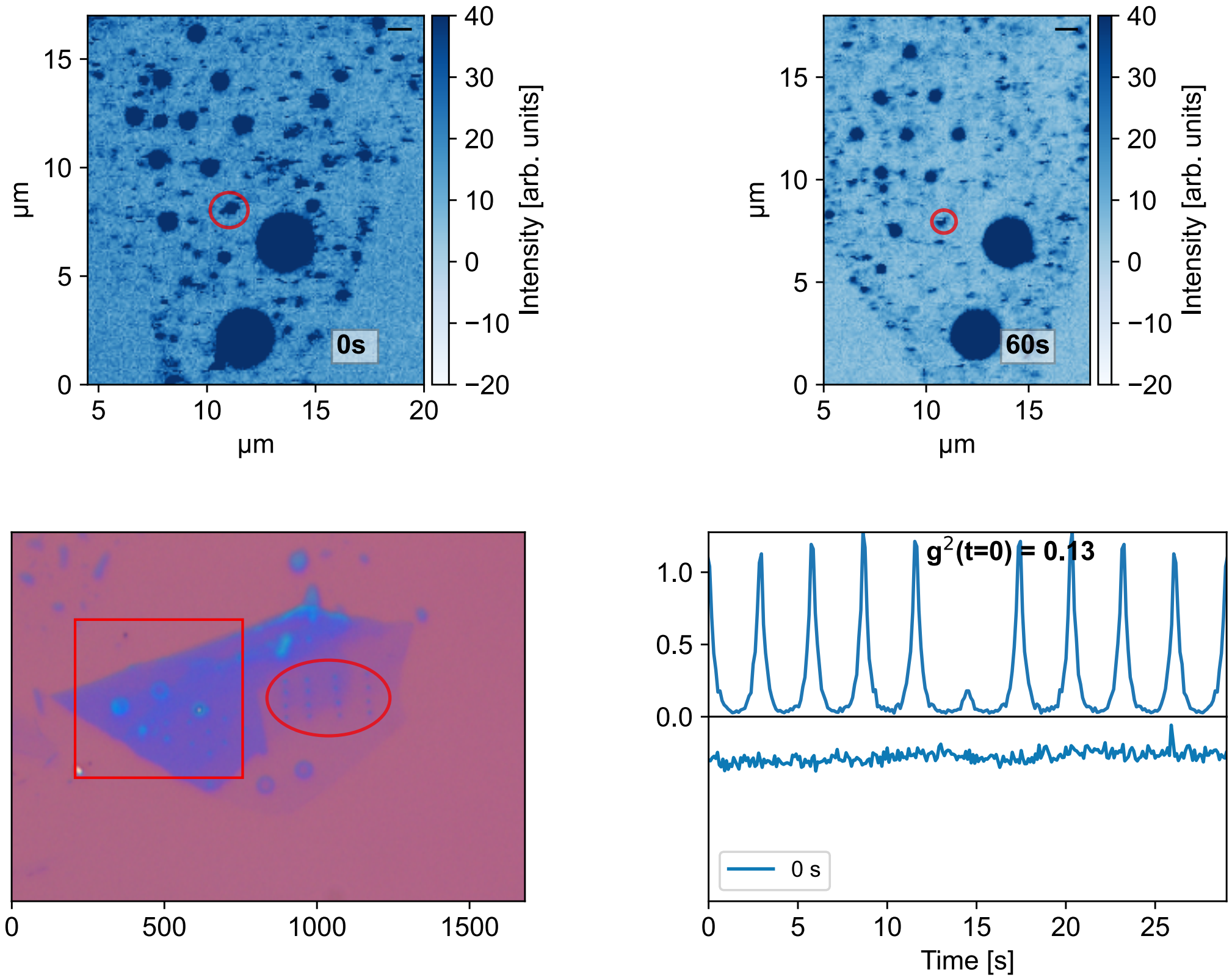

Figure S11: Confocal PL maps acquired before (a) and after (b) 60s of plasma etching. (c) Optical microscope image of the hBN flake on a SiO_2/Si substrate, with the square indicating the region where the PL maps were acquired. (d) Second-order autocorrelation function, $g^{(2)}(\tau)$, and (e) time-resolved PL measurement of the emitter marked by the red circle in (a), located several micrometers away from the electron-irradiated region.

References

[1] A. Kumar, C. Samaner, C. Cholsuk, T. Matthes, S. Paçal, Y. Oyun, A. Zand, R. J. Chapman, G. Saerens, R. Grange, et al., *ACS Nano* **2024**, *18*, 7 5270.

[2] A. Kumar, C. Cholsuk, A. Zand, M. N. Mishuk, T. Matthes, F. Eilenberger, S. Suwanna, T. Vogl, *APL Mater.* **2023**, *11*, 7 071108.

[3] S. Nedić, K. Yamamura, A. Gale, I. Aharonovich, M. Toth, *Adv. Opt. Mater* **2024**, *12*, 24 2400908.

[4] M. Hazra, M. Rieger, A. Kumar, M. N. Mishuk, C. Cholsuk, K. Sripathy, V. Villafañe, K. Muller, J. J. Finley, T. Vogl, *ACS Photonics* **2026**, *13*, 4 1176.

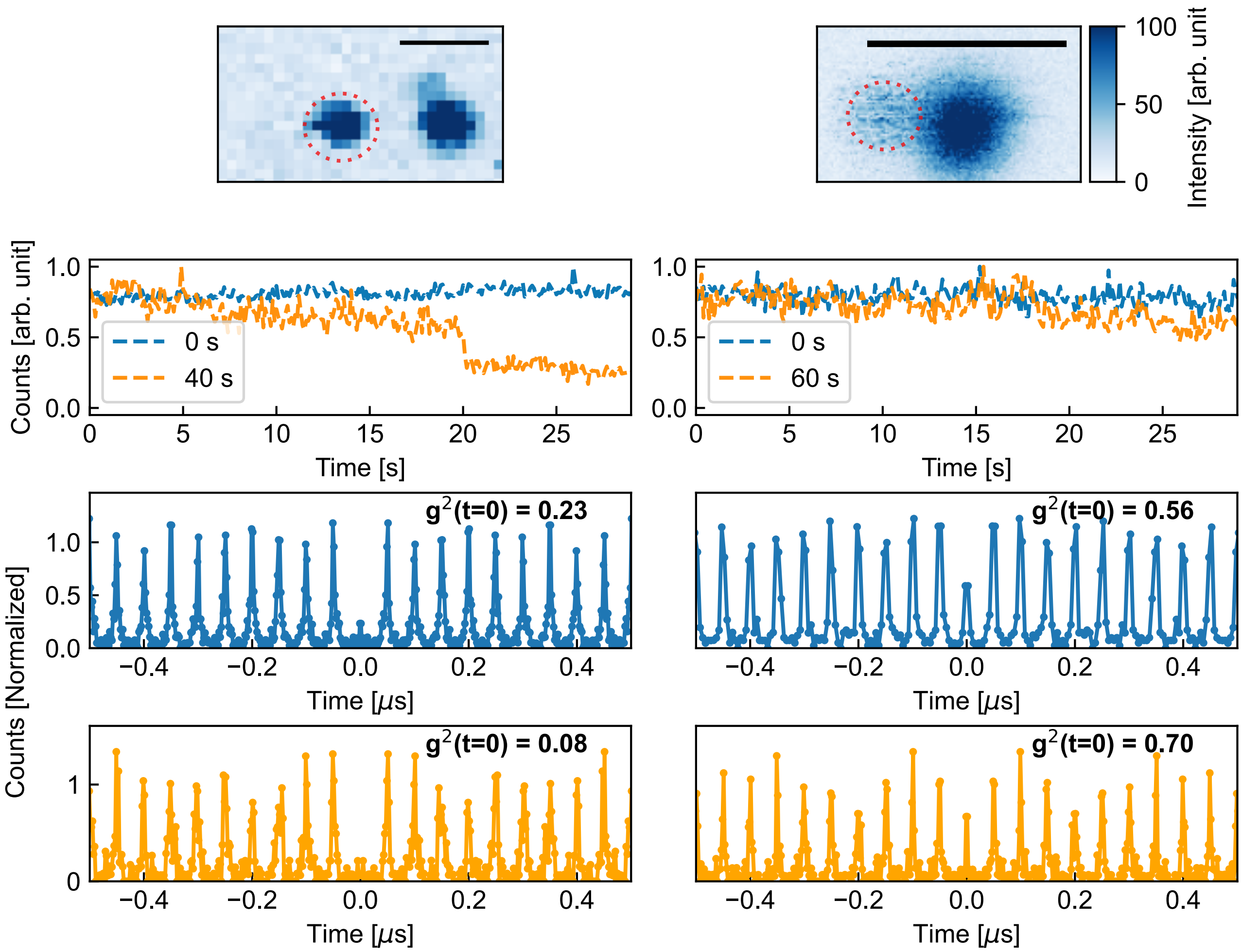

Figure S12: Photophysical properties of two representative emitters in the hBN flake shown in a circle in Figure. **S11**(c). Each column corresponds to an emitter, indicated by red dotted circle in the PL intensity map shown in the top row. From top to bottom: PL intensity map, normalized PL spectrum, and second-order autocorrelation function, $g^{(2)}(\tau)$, of the same emitter measured before and after plasma etching. The blue and orange curves represent the measurements acquired before and after etching, respectively. The corresponding plasma etching duration is indicated in the PL spectrum for each emitter.

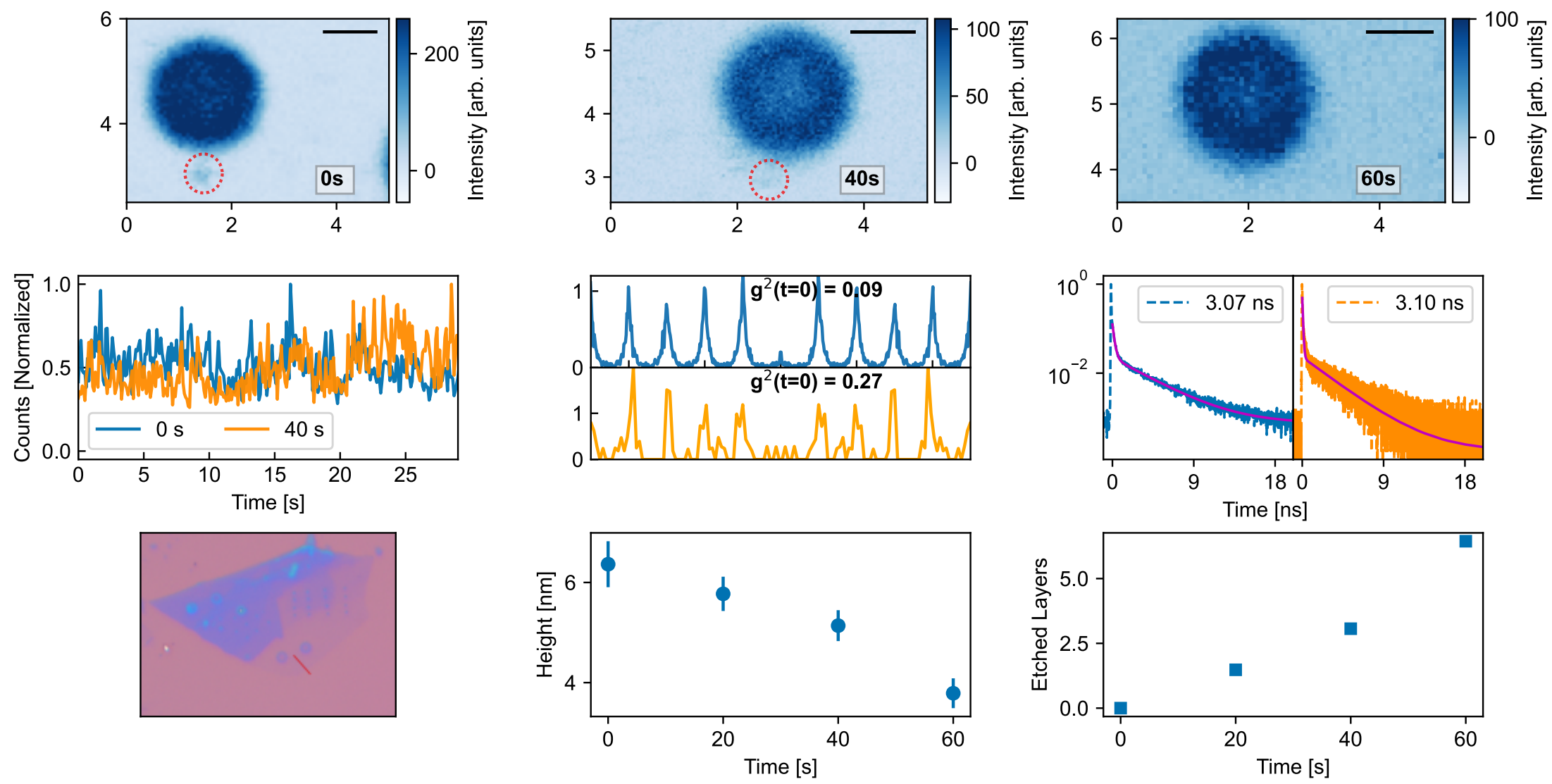

Figure S13: Photophysical characterization of a green–yellow emitter initially located in a 6.4 nm-thick hBN flake. Confocal PL maps acquired after successive plasma-etching steps: before etching (a), after 40 s (b), and after 60 s (c). The emitter disappeared after 60s etching. (d) Time-resolved PL, (e) $g^{(2)}(\tau)$, and (f) PL lifetime of the emitter before (blue) and after 40 s of plasma etching (orange). (g) Optical microscope image of the hBN flake, with the line indicating the location of the AFM line profile. (h) AFM thickness acquired after successive plasma-etching steps, showing the flake thinning. (i) Number of hBN layers removed from the top surface as a function of plasma-etching time.

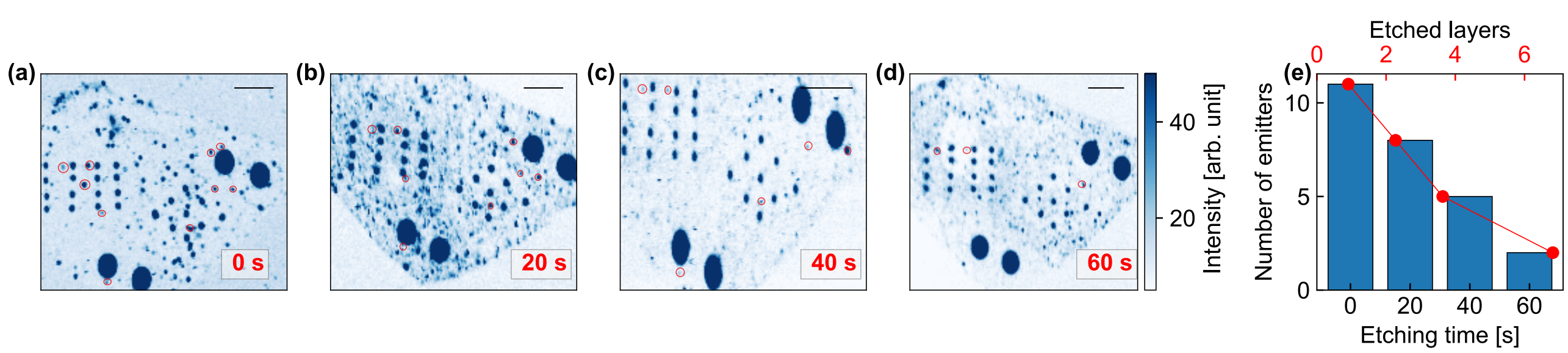

Figure S14: PL intensity maps at different etching times. (a–d) Maps acquired before etching and after oxygen plasma etching for 20, 40, and 60 s, respectively. (e) Number of emitters as a function of etching time. Only emitters that were present prior to etching are considered, as oxygen plasma treatment can also generate additional emitters. The values represent a rough estimate, since not all emitters across the flake were systematically analyzed. The emitters that were analyzed before plasma etching are marked in red circles in (a–d). Scale bars correspond to 5 μm.
